# Snapshot of the Eukaryotic Gene Expression in Muskoxen Rumen—A Metatranscriptomic Approach

**DOI:** 10.1371/journal.pone.0020521

**Published:** 2011-05-31

**Authors:** Meng Qi, Pan Wang, Nicholas O'Toole, Perry S. Barboza, Emilio Ungerfeld, Mary Beth Leigh, L. Brent Selinger, Greg Butler, Adrian Tsang, Tim A. McAllister, Robert J. Forster

**Affiliations:** 1 Lethbridge Research Centre, Agriculture and Agri-Food Canada, Lethbridge, Alberta, Canada; 2 Department of Biological Sciences, University of Lethbridge, Lethbridge, Alberta, Canada; 3 Centre for Structural and Functional Genomics, Concordia University, Montreal, Quebec, Canada; 4 Department of Biology and Wildlife, Institute of Arctic Biology, University of Alaska Fairbanks, Fairbanks, Alaska, United States of America; Max Planck Institute for Evolutionary Anthropology, Germany

## Abstract

**Background:**

Herbivores rely on digestive tract lignocellulolytic microorganisms, including bacteria, fungi and protozoa, to derive energy and carbon from plant cell wall polysaccharides. Culture independent metagenomic studies have been used to reveal the genetic content of the bacterial species within gut microbiomes. However, the nature of the genes encoded by eukaryotic protozoa and fungi within these environments has not been explored using metagenomic or metatranscriptomic approaches.

**Methodology/Principal Findings:**

In this study, a metatranscriptomic approach was used to investigate the functional diversity of the eukaryotic microorganisms within the rumen of muskoxen (*Ovibos moschatus*), with a focus on plant cell wall degrading enzymes. Polyadenylated RNA (mRNA) was sequenced on the Illumina Genome Analyzer II system and 2.8 gigabases of sequences were obtained and 59129 contigs assembled. Plant cell wall degrading enzyme modules including glycoside hydrolases, carbohydrate esterases and polysaccharide lyases were identified from over 2500 contigs. These included a number of glycoside hydrolase family 6 (GH6), GH48 and swollenin modules, which have rarely been described in previous gut metagenomic studies.

**Conclusions/Significance:**

The muskoxen rumen metatranscriptome demonstrates a much higher percentage of cellulase enzyme discovery and an 8.7x higher rate of total carbohydrate active enzyme discovery per gigabase of sequence than previous rumen metagenomes. This study provides a snapshot of eukaryotic gene expression in the muskoxen rumen, and identifies a number of candidate genes coding for potentially valuable lignocellulolytic enzymes.

## Introduction

Within the gastrointestinal tract of herbivores a complex group of anaerobic microorganisms, including bacteria, archaea and eukaryotes, produces a vast array of lignocellulolytic enzymes that in turn digest complex plant cell wall polysaccharides and ferment the released simple sugars. The resulting volatile fatty acids and microbial protein are a source of carbon, nitrogen and energy for the host [1], [2]. Substantial efforts have been made to understand polysaccharide digestion within the rumen through isolation and identification of cellulolytic species, characterization of their enzymes [3], and sequencing the genomes of the major culturable rumen bacteria [4], [5], [6], [7], [8], [9]. The recent introduction of massively parallel sequencing technologies has enabled the sequencing of herbivore gut microbiomes, including foreguts of cattle and wallabies [10], [11], [12] and the hindgut of termites [13]. These studies have led to the identification of novel cellulolytic enzymes, many of which quite likely arise from the large majority of environmental microbes that are unculturable in the laboratory [4].

Despite the prolific activity directed at understanding the rumen microbiome, there is a distinct lack of information about the eukaryotic component of the rumen metagenome and no rumen fungal or protozoal genomes have been reported. Only a small portion of genes described in previous metagenomic studies were attributed to eukaryotes [10], [11], [12], although the role of fungi (Neocallimastigomycota) and protozoa (Litostomatea) in rumen cellulose digestion is widely recognized [14], [15]. The lack of genomic information about anaerobic eukaryotes in the rumen is likely a consequence of 1) the low abundance of eukaryotic DNA in the rumen metagenome; 2) the inadvertent exclusion of eukaryotic species by sample preparation methods; and 3) the failure of bioinformatic analyses to annotate novel eukaryotic gene sequences.

Rumen anaerobic fungi not only produce highly active fibrolytic enzymes targeting the plant cell walls, but they also physically disrupt plant cell walls including the cuticle via penetrating rhizoids. Zoospores, the mobile phase of the fungal life cycle, also preferentially colonize lignin-rich regions of the plant cell wall and upon germination, solubilize these regions to a greater extent than rumen bacteria. Studies have shown that rumen fungi may account for up to 8∼20% of the total rumen microbial biomass in ruminants consuming forage [14], [16]. A recent study demonstrated that anaerobic fungi are widely distributed in both ruminant and non-ruminant herbivores [17].

The effects of rumen protozoa on fiber digestion are less clear. Previous studies are inconsistent and reports on the effects of protozoa range from an increase of fibre digestion by 50% to a decrease of 15% (for review, see [15]). Studies on the contribution of rumen protozoa to fiber degradation have also been hampered by the difficulty in cultivating protozoa *in vitro* without the presence of associated bacteria. However, glycoside hydrolase activities and genes have been identified from these organisms [18], [19].

Identification of potent lignocellulolytic and other carbohydrate active enzymes are of great interest for industrial processes, such as cellulosic ethanol production [20], [21]. In the present study we applied mRNA-Seq technology [22] to target the polyadenylated eukaryotic mRNA from the muskoxen (*Ovibos moschatus*) rumen microbial consortium. Muskoxen are arctic ruminants that live primarily in northern Canada, Alaska and Greenland. For the majority of the year, their food sources are limited to forages high in lignocellulose content, due to the very short arctic summer [23]. Consequently, we speculated that muskoxen have evolved to harbour a microbial community that efficiently degrades plant cell wall fiber [24]. The sampled muskoxen were maintained at an isolated wildlife research facility (R.G. White Large Animal Research Station, University of Alaska Fairbanks, Fairbanks, AK) and had not been in contact with domestic ruminants. It was hypothesized that the metatranscriptome approach would lead to the identification of genes coding for novel enzymes and also, for the first time, provide information on the expression of carbohydrate active enzymes by the eukaryotic community in the rumen.

## Results

### General sequence statistics

**Figure 1:**
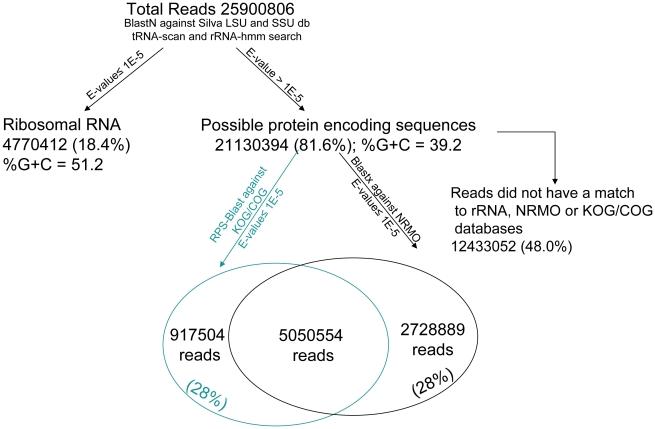
Summary statistics for muskoxen rumen eukaryotes metatranscriptome analysis.

In the present study, we adopted a metatranscriptomic approach to identify enzymes from the muskoxen rumen microbial community. We used samples from muskoxen fed on triticale straw and brome hay for deep sequencing with the goal to obtain transcripts encoding carbohydrate-active enzymes. Total RNA was extracted from rumen solids to ensure isolation of the cellulolytic microbial biofilm as well as RNA from microbes deeply embedded in the plant fiber. After purification, the eukaryotic mRNA was sequenced using the Illumina Genome Analyzer II platform. This approach resulted in a total of 25,900,806 reads, with an average read length of 108 nt, generating a total of 2.8 gigabases of sequence data (Figure 1).

Raw sequencing reads were assembled into a total of 59,129 contigs with an average length of 310 bp, for a total of 19 M base pairs. The maximum length of the contigs was 13,498 bp, which contained a single open reading frame of 13,083 bp. It encoded for a 4354-amino-acid protein that showed 28% identity to a hypothetical protein from a strain of *Orpinomyces* [25]. The size distribution of all the contigs is illustrated (Figure S1). Over 12,000 contigs were represented by 100 or more reads, including 2551 contigs represented by more than 1000 reads and 545 represented by more than 5000 reads. To validate the contig assembly, twenty glycoside hydrolase related contigs (≥500 bp) were chosen and primers designed to amplify the target [Table S2]. Nineteen of the twenty contigs were successfully amplified using at least one set of primers.

Using BLASTN searches against the Silva non-redundant large subunit (LSU) and small subunit ribosome (SSU) ribosomal RNA databases coupled with rRNA-HMM and tRNA-scan, we identified 4.77 million non-coding (nc) RNA reads or 18.4% of the total (Figure 1). During the RNA sample preparation step, oligo-dT coated magnetic beads were used to remove a large proportion of rRNA. Assuming ncRNA account for 95% of the original total RNA [26], approximately 99% of this amount was successfully removed as indicated by the percentage of the ncRNA reads identified.

The average GC content of ncRNA reads was 51%. In contrast, the GC content of all potential protein encoding RNA reads was 39.2%, a value that was significantly lower than the ncRNA reads and much lower than those reported in metagenomic studies of other microbial communities associated with ocean, fresh water and various animal environments (average %GC 49.56±4.9 [27]). The average GC content of the contigs was 37.9%, which were also lower than other metagenomic studies (Figure S2).

BLASTX searches using the 21 million potential protein encoding reads against the trimmed down non-redundant amino acid database (NRMO) from Genbank indicated 7.8 million reads had at least one significant match (E-value ≤10^−5^). RPS-BLAST searches against the euKaryotic Orthologous Groups (KOG) and the Cluster of Orthologous Groups (COG) identified 6.0 million reads that may have conserved domains (Figure 1), of which 0.9 million reads were not identified by BLASTX searches. The remaining reads (48% of all reads) did not show any similarity to any of the above mentioned databases.

**Figure 2:**
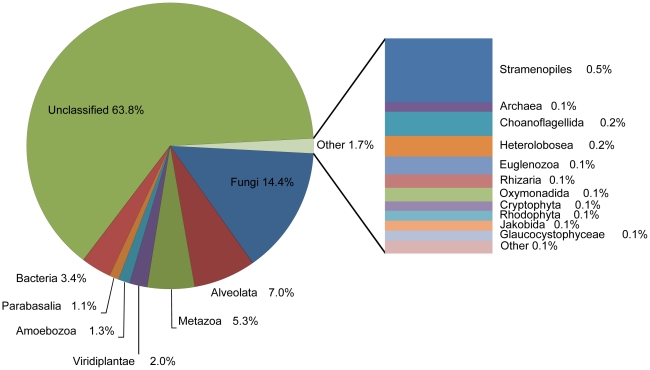
Phylogenetic distribution of muskoxen rumen metatranscriptome putative protein encoding reads (a total of 21.1 million) based on MEGAN analysis of top BLASTX hits against the NRMO database. The percentages of the major phylogenetic groups were indicated.

The first BLASTX match was used to estimate the possible origin of each putative protein coding RNA reads, according to MEGAN analysis (Figure 2). About 6.6 million reads (31%) showed highest similarity to proteins from the Eukaryota kingdom. Among these, Rumen anaerobic fungi, which belong to the phylum Neocallimastigomycota [28], were represented by 1.4 million reads. Rumen ciliate protozoa, which belong to the Litostomatea class of Alveolata group, were represented by 1.1 million reads. At the genus level, the most represented genera were known rumen/anaerobic species, including *Entodinium*, *Piromyces*, *Neocallimastix*, *Trichomonas*, *Orpinomyces*, *Entamoeba*, and *Epidinium*, that were represented by over 100,000 reads each (Figure S3). A total of 0.7 million reads (3.4%) and 0.02 million reads (0.1%) were binned to Bacteria and Archaea kingdoms, respectively.

### Analysis of sequencing coverage

The sequencing coverage was first assessed by looking at the matches to rumen eukaryotic proteins that were present in the nr database. Rumen anaerobic fungal protein sequences (total of 257) were obtained from the Genbank database as of July, 2010. TBLASTN searches were performed using all of these proteins as queries, and matches to 220 of them (with Expect value ≤1E-20) were found in the muskoxen contigs. Another similar search identified 104 of 107 published rumen protozoal protein sequences in our dataset. The identification of almost all known rumen fungal and protozoal sequences demonstrated the comprehensive coverage of the current sequencing approach.

**Figure 3:**
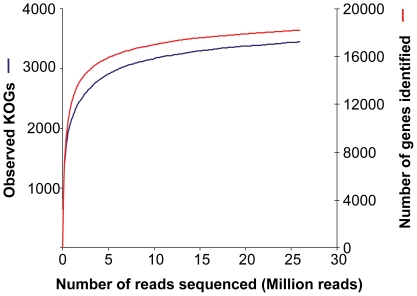
Collector's curve of gene richness as a function of reads analyzed.

The coverage was further evaluated by plotting collector's curves based on the number of functional gene categories (matched to the KOG database) and gene accession numbers identified (matched to the NRMO database), as a function of the number of reads (Figure 3). Saturating coverage was found for both curves, as roughly 80% of the total richness was found with less than 14% of the sequencing effort.

### Functional analysis of the putative protein-coding reads

Based the RPS-BLAST search results, 6.0 million reads could be assigned to a cluster of the KOG/COG databases. Most of the assignable sequences belonged to the “Translation, ribosomal structure and biogenesis” cluster (45% of all the assigned sequences), while Cytoskeleton (16%) was the second largest cluster. Correspondingly, 9 of the top 10 KOG/COGs also belonged to these two categories, with KOG0676 (Actin and related proteins, which were represented by 551,126 reads) and KOG0052 (Translation elongation factor one, 230,087 reads) as the first two abundant KOG groups (Figure S4). These results indicate that these groups of genes were actively transcribed. About 18% of the reads that matched the KOG/COG databases were involved in Metabolism, including metabolism of carbohydrate (7.9%), energy conversion (4.2%) and amino acids (2.6%). KOG/COG groups involved in the glycolysis and pyruvate metabolism were represented by highest read numbers (Table S3), demonstrating genes belonging to those clusters had a central role in the metabolic pathway.

Hydrogenosomes are membrane-bound organelles present in anaerobic eukaryotic species such as rumen fungi *Neocallimastix* and *Piromyces* as well as rumen protozoa including *Eudiplodinium maggii* and *Dasytricha ruminantium* [29], [30]. Hydrogenosomes are distantly related to mitochondria and are the centre of ATP and hydrogen generation in these microorganisms [30]. In the present rumen metatranscriptome dataset, KOG/COGs linking with hydrogenosomes were also identified, including iron-only hydrogenase, malic enzyme, pyruvate:formate lyase, succinyl-CoA synthetase and acetate:succinate CoA-transferase. Combining the highly expressed KOG/COGs, a representative energy metabolism pathway of muskoxen rumen eukaryotes was reconstructed (Figure S5).

### Lignocellulolytic enzymes

**Figure 4:**
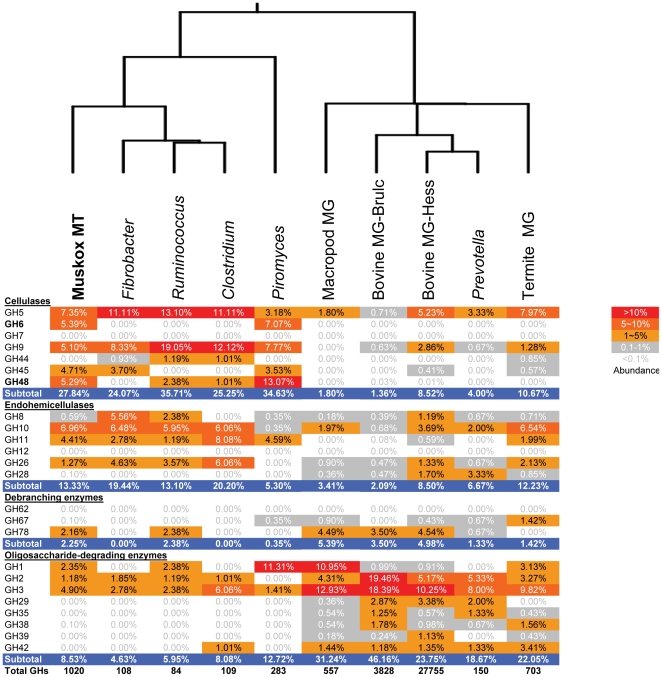
Comparison of the carbohydrate active enzymes identified from muskoxen rumen metatranscriptome (using all assembled contigs) with those of four other foregut metagenomes and five rumen/anaerobic microorganisms. The percentages of each enzyme family were shown in the cells. Refer to Table S4 for a complete comparison. Dendrogram on the top indicates the relationship of the GHs identified based on similar percentage distribution. **Muskox MT**: Muskoxen rumen metatranscriptome; **Fibrobacter**: Genome of *Fibrobacter succinogenes* S85; **Ruminococcus**: Genome of *Ruminococcus flavefaciens*; **Clostridium**: Genome of *Clostridium thermocellum*; **Piromyces**: EST sequence of *Piromyces* sp. E2; **Macropod MG**: Macropod foregut microbiome [11]; **Termite MG**: Termite hindgut microbiome [13]; **Bovine MG-Hess**: Bovine Rumen microbiome by Hess et al [12]; **Bovine MG-Brulc**: Bovine Rumen microbiome by Brulc et al [10]; and **Prevotella**: Genome of *Prevotella ruminicola*.

Sequence homology based enzyme annotation was biased toward the identification of known enzymes that were already present in the database. To minimize this bias, we used a more sensitive Pfam-HMM search to identify Carbohydrate Active enZYme (CAZy) modules (Figure 4, Table 1, Table S4). In all, these analyses identified 400 k reads potentially encoding lignocellulolytic enzyme modules, spanning about 110 CAZy families. The read number in each family gives an indication of the expression status of that group. However, it needs to be pointed out that the number of reads that matched to lignocellulolytic enzymes was likely underestimated because the short reads (108 nt, translated into 36 amino acid residues maximum) resulted in a less robust database search score than that which would have been obtained using the full sequence of complete genes.

**Table 1:** List of putative carbohydrate esterases, pectate lyases and carbohydrate binding related modules in the muskoxen rumen eukaryotic metatranscriptome (Muskoxen MT), and comparison of their abundance of selected GH and CBM domains with those of three other foregut microbiomes: foregut of wallaby (Macropod); bovine rumen (Bovine 2009 and Bovine 2011) and hindgut of termite (Termite).

	MuskOx Contigs ≥500 bp	Macropod	Bovine 2009	Bovine 2011	Termite
***Carbohydrate Esterases***					
CE1	13	0	11	NR	NR
**CE2/CE3**	**6**	**0**	**1**	**NR**	**NR**
CE4	50	24	4	NR	NR
**CE6**	**0**	**0**	**0**	**NR**	**NR**
CE7	19	3	2	NR	NR
CE8	0	3	0	NR	NR
CE9	3	14	0	NR	NR
CE10	3	18	0	NR	NR
CE11	4	2	0	NR	NR
CE12	0	6	0	NR	NR
CE13	5	0	NR	NR	NR
CE14	0	1	NR	NR	NR
**CE15**	**0**	**0**	**NR**	**NR**	**NR**
**CE16**	**8**	**0**	**NR**	**NR**	**NR**
***Pectate Lyase***					
**PL1**	**6**	**1**	**0**	**NR**	**NR**
PL2	0	0	0	NR	NR
**PL3**	**4**	**0**	**0**	**NR**	**NR**
PL5	0	0	0	NR	5
PL6	7	0	0	NR	NR
**PL9**	**8**	**0**	**0**	**NR**	**NR**
PL10	0	0	0	NR	NR
**PL11**	**1**	**0**	**0**	**NR**	**5**
PL12+15+17+21	0	0	NR	NR	NR
***Carbohydrate Binding Modules***					
**CBM1**	**33**	**0**	**0**	**0**	**0**
CBM2	0	1	0	50	0
CBM3	3	0	0	33	0
CBM4/9/16/22	3	6	0	417	5
CBM6	27	3	0	932	13
**CBM10**	**403**	**0**	**0**	**1**	**0**
CBM11	0	0	0	NR	3
CBM13	31	2	1	118	0
**CBM18**	**108**	**0**	**0**	**0**	**0**
CBM20	5	3	0	112	0
CBM21	0	1	0	1	0
CBM26	0	1	0	NR	0
**CBM29**	**11**	**0**	**0**	**NR**	**0**
CBM30	0	0	0	NR	1∼8
CBM32/47	2	6	1	747	4
CBM34	0	2	0	72	0
CBM35	1	0	0	NR	0∼1
CBM36	8	0	0	NR	2∼13
CBM37	3	0	0	NR	1
CBM48	3	22	0	NR	0∼1
CBM50	11	47	NR	1887	NR
CBM51	0	1	NR	173	NR

Domains identified from muskoxen MT contigs that significantly different from the rest data are indicated in bold.

**Figure 5:**
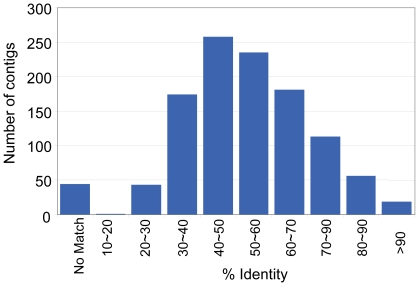
Amino acid sequence similarities of carbohydrate active enzymes identified from the muskoxen rumen metatranscriptome (using all assembled contigs) versus top BLASTX match to the Genbank nr database.

To circumvent this problem, similar analyses were also performed on the assembled contigs. In total, we identified over 2500 contigs with a significant match to at least one CAZy module (Table S4). Since the use of short contigs may overestimate the total number of enzymes, we further restricted our targets to those contigs longer than 500 base pairs (1082 in total, Tables S4, S5, S6). These contigs, especially those assembled from larger numbers of reads, could serve as good candidates of potentially useful lignocellulolytic enzymes. Only 17% of these contigs were more than 70% identical to genes in the nr database, while 46% of them had less than 50% identity (Figure 5). Seventeen percent of the CAZy genes identified were most similar to nr database sequences annotated as “(Conserved) hypothetical protein”, “predicted protein” or “unnamed protein product”. These results indicated that rumen eukaryotes produce a large variety of glycoside hydrolases (GHs) with many of them remaining uncharacterized. There were 242 contigs that had two or more distinct CAZy domains. Carbohydrate binding domain (CBM) family 10/fungal dockerin domains were predominant in these multi domain enzymes, and were found in 190 (78.5%) of these contigs. Glycoside hydrolases from families GH6, GH45, and GH48 were found in 25, 25 and 23 multi-domain contigs respectively, most of which were linked to CBM10 modules.

#### Catalytic modules

Most of the muskoxen rumen microbiome cellulases identified were classified as families GH5, 6, 9, 45 and 48, which were represented by 30 to 51 contigs. Similar to other rumen metagenomic studies [10], [11], [12], no contigs showed similarity to family GH7 or GH44. In this study, putative swollenin modules were detected in 16 contigs. Swollenin was reported to dissociate cellulose fiber with no hydrolytic activity [31] and has not been previously reported to be associated with anaerobic microorganisms. Hemicellulose degrading enzymes from GH8, GH10, GH11, GH26 and GH53 were also identified. GH10 and GH11 were the predominant families that were represented by 29 and 33 contigs, respectively. Carbohydrate esterases remove the ester bond on the xylan backbone, exposing it to glycoside hydrolases. There were 111 contigs showing similarity to carbohydrate esterases in the CAZy database. Carbohydrate esterase family 4 (CE4) was the largest family with 50 contigs. The CE1 family, encoding feruloyl esterases important for lignin solubilization in the rumen, was represented by 13 contigs. Twenty seven contigs were annotated to be polysaccharide lyases (Table S4).

GH family profiles recovered from the metatranscriptome of muskoxen rumen were compared to the metagenomes of the termite hindgut [13], wallaby foregut [11] and bovine rumen [10], [12], as well as the genomes of several rumen anaerobic bacteria and expressed sequence tags (ESTs) of the rumen fungi *Piromyces* sp. E2 (Figure 4 and Table S4). Clustering analysis of the GH family distribution (Figure 4) showed that the muskoxen metatranscriptome was most closely related to rumen cellulolytic bacteria and the rumen fungus *Piromyces*. There were some major differences between genes identified by our study and previous metagenome sequencing studies. Approximately 28% of identified glycoside hydrolases were cellulases in the muskoxen rumen transcriptome, as opposed to 8.5% or less in reported rumen metagenome studies. A large number of genes potentially encoding GH6, GH48 and swollenin enzymes identified in the present study were rarely found in other studies of rumen metagenomes. Conversely GH29, 35, 39 and 42 family members described in other rumen metagenome studies [10], [11], [12] were not identified in the rumen metatranscriptome of muskoxen (Figure 5, Table S4).

#### Accessory modules

The most abundant accessory module was the carbohydrate binding module of family 10 (CBM10), which was identified from 403 contigs that longer than 500 bp (Table 1). CBM10 has been shown to be associated with many rumen fungal glycoside hydrolases. It was once proposed to be a eukaryotic counterpart of bacterial cellulosomal dockerin [32], but a recent study [33] suggested that the function of this domain differs from bacterial dockerins. Dockerin containing cellulases are known to interact with cellulosome scaffolding proteins in bacteria to form the cellulosome structure. However, a cellulosome scaffolding protein has yet to be identified from rumen fungi. The exact function of CBM10 modules remain to be explored.

CBM18, known as a chitin binding module, was the second largest CBM group, and was identified from 108 contigs. Other major CBMs identified included CBM1, CBM6 and CBM13, that presented in 33, 27 and 31 contigs, respectively. Accessory modules that are commonly found in bacterial cellulases, such as bacterial cellulosome dockerin, cohesin, S-layer homolog domain (SLH), cellulase N-terminal immuno-globulin domain (CelD_N) and fibronectin-3 (fn3) modules, were not found in the contigs or reads.

#### Lignocellulolytic gene expression

Both gene diversity and gene expression information can be obtained from the metatranscriptomic sequencing analysis. To demonstrate the latter, we summarized the read numbers associated with contigs/genes (Tables 2, S5, S6). Cellulase and xylanase genes in families 6, 11, 45 and 48 were over-represented compared to other families (Table 2). GH1 genes, which encode oligosaccharide degrading enzymes, were also over-represented. In addition, a putative family 6 polysaccharide lyase, which has never been reported from eukaryotes, was represented by over 20 k reads.

**Table 2:** Carbohydrate active enzymes identified with 10,000 or more reads in the metatranscriptome from muskoxen rumen.

Contig	Domains	Length	No. of Reads	E-value	Id%	BLASTX Hit Description
Contig21589	Glyco_hydro_48; CBM_10	2292	52778	0	78.2	cellulase Cel48A precursor [Piromyces sp. E2]
Contig28863	Glyco_hydro_11	1148	44380	0	86.8	xylanase [Neocallimastix frontalis]
Contig22047	Glyco_hydro_6; CBM_10	1921	44342	0	83.1	cellobiohydrolase II-like cellulase CelH [Orpinomyces sp. PC-2]
Contig28684	Glyco_hydro_48	898	33408	1.00E-154	81.1	cellulase Cel48A precursor [Piromyces equi]
Contig627	CBM_20; PL6	1777	20162	7.00E-14	24.5	Parallel beta-helix repeat protein [Planctomyces maris DSM 8797]
Contig27493	Glyco_hydro_16	878	19963	9.00E-74	57.1	Licheninase [Fibrobacter succinogenes subsp. succinogenes S85]
Contig28658	Glyco_hydro_48; CBM_10	1093	16001	1.00E-145	74.3	cellulase Cel48A precursor [Piromyces sp. E2]
Contig21206	Glyco_hydro_45	925	15508	1.00E-68	52.4	Cellulase [Fibrobacter succinogenes subsp. succinogenes S85]
Contig21311	GH94; GT36_AF	2521	15361	0	72.5	cellobiose phosphorylase [Prevotella ruminicola 23]
Contig21506	Glyco_hydro_1	821	14352	1.00E-110	88.6	beta-glucosidase Cel1C [Piromyces sp. E2]
Contig29533	Glyco_hydro_1	819	14351	1.00E-138	88.2	beta-glucosidase [Orpinomyces sp. PC-2]
Contig3078	Glucosaminidase	601	13803	1.00E-26	72.5	Muramidase (flagellum-specific) [Eubacterium rectale A1-86 (DSM 17629)]
Contig29741	Glyco_hydro_1	586	13272	7.00E-91	84.7	beta-glucosidase Cel1C [Piromyces sp. E2]
Contig29986	CBM_1	854	13133	6.00E-33	37.8	Xylanase B; XYLB [Neocallimastix patriciarum]
Contig29325	Glyco_hydro_43	1409	12451	1.00E-113	59.0	beta-xylosidase/alpha-L-arabinfuranosidase, putative, gly43F [Cellvibrio japonicus Ueda107]
Contig26982	Glyco_hydro_1	1879	11902	0	82.3	beta-glucosidase [Orpinomyces sp. PC-2]
Contig12311	Glyco_hydro_1	1369	11613	0	83.7	beta-glucosidase [Piromyces sp. E2]
Contig30005	Glyco_hydro_48	1203	11562	0	83.1	cellulase Cel48A precursor [Piromyces sp. E2]
NODE_3576	Glyco_hydro_48; CBM_10	2067	11488	0	79.8	cellulase Cel48A precursor [Piromyces sp. E2]
Contig29850	Glyco_hydro_48; CBM_10	1092	11311	1.00E-152	73.8	cellulase Cel48A precursor [Piromyces equi]
Contig30163	Glyco_hydro_1	954	11088	1.00E-146	74.1	beta-glucosidase [Orpinomyces sp. PC-2]
Contig30798	Glyco_hydro_45	523	10896	3.00E-48	63.6	Cellulase [Fibrobacter succinogenes subsp. succinogenes S85]
Contig29098	Glyco_hydro_48; CBM_10	1484	10522	1.00E-145	71.3	cellulase Cel48A precursor [Piromyces equi]
Contig1597	Glyco_hydro_6; CBM_10	1020	10347	1.00E-117	65.4	exoglucanase Cel6A [Piromyces sp. E2]
Contig27001	Glyco_hydro_43	1127	10074	1.00E-115	60.1	beta-xylosidase/alpha-L-arabinfuranosidase, putative, gly43F [Cellvibrio japonicus Ueda107]

## Discussion

Cellulase activities are well known to be present in microbial communities found in soil, compost, and herbivore digestive tracts including the rumen. Metagenomic technology coupled with high throughput sequencing has enabled the identification of thousands of genes encoding for enzymes that degrade plant cell walls [10], [12], [34]. In the present study, we used rumen solid samples from muskoxen fed a highly lignified diet of triticale straw or brome grass hay. Rumen solids are reportedly responsible for 80∼90% of the endoglucanase and xylanase activities in the rumen [35], [36], as attachment and the formation of digestive microbial biofilms is a prerequisite for the ruminal digestion of feed [35]. By applying an improved isolation method, high quality RNA was extracted from the rumen solids [37]. Combined with an efficient method of *de novo* assembly of short sequence reads, the present study has provided a comprehensive catalogue of eukaryotic cellulolytic enzymes in the muskoxen rumen, many of which are supported by full-length cDNA information.

To our knowledge, we are the first group to report the whole eukaryotic transcriptome of a rumen microbial community. Metatranscriptomic studies have been previously carried out in various microbiomes focused on marine microbial communities [38], [39], [40], [41], and gut microbial population of the piglet [42]. Most of these studies used Roche 454 pyrosequencing technology and although the read lengths were longer than that obtained with Illumina sequencing, far fewer total sequences were produced. In fact most of these studies produced less than 500 M bp of sequences and sample-sequencing depth was low [41]. Illumina sequencing in the present study generated 2.8 gigabases of sequencing data, which is at least 6 times that of previous metatranscriptomic studies [38], [39], [40], [41], [42]. The deep sequencing coverage obtained in this study is attested by the observation that the slopes of the collector's curves reached a plateau and that most of the rumen eukaryotic sequences reported in Genbank were identified in the metatranscriptome (331 out of 364 sequences).

Metatranscriptomics has distinct properties when compared to metagenomics, the first being that metatranscriptomic analysis identifies most extensively transcribed genes while metagenomic sequencing identifies the most numerically dominant genes. For example, *Prevotella* is a group of predominant rumen bacteria that at times account for as much as 60% of the total bacteria in the rumen [43], [44], even though they play no active role in the digestion of recalcitrant cellulose [8]. Indeed, the GH profiles of *Prevotella ruminicola* clustered closely to those of the wallaby and bovine rumen metagenomes, with GHs involved in the degradation of oligosaccharides and hemicelluloses being highly represented, whereas the proportion of GHs related to cellulase were much lower (Figure 4). If a gene encoding a GH was present in a microbial species of low rumen abundance, even if highly expressed, it would be less likely to be recovered by metagenomic sequencing. Such a scenario may be applicable to the rumen anaerobic fungi as they are reported to account for no more than 8% of the total rumen microbial biomass [45]. Secondly, metatranscriptomic analysis may provide insight into the degree of gene expression (Table 2), which could help focus gene mining towards those enzymes that are most active in plant cell wall degradation. Consequently, the yield of potential gene targets for further development may be far higher with metatranscriptomic than metagenomic approaches. A recent rumen metagenomics study using Illumina sequencing technology generated 268 gigabases of metagenomic DNA with about 103 putative carbohydrate active enzymes identified per gigabase [12]. In our present study, we were able to identify 2500 genes in 2.8 gigabases of RNA sequence, translating to 893 putative carbohydrate active enzymes per gigabase.

When muskoxen are fed on high fiber diets, cellulolytic microorganisms attach to and penetrate the fiber and expression of many of their cellulolytic enzyme genes is induced. Genes that are highly expressed generate more sequencing reads, increasing sequence coverage, resulting in the assemblage of longer contigs and thereby increases the likelihood of regenerating full length genes or modules. Indeed, of the 59129 contigs in the present study, there were over 2500 contigs with lengths over 1.0 kb and 6022 with lengths between 0.5 to 1 kb (Figure S1). Among the contigs longer than 500 bp, over 1000 of these harboured genes putatively encoding for carbohydrases. Most CAZy genes longer than 500 bp (96%) were represented by 100 or more reads (Table S5/S6), corresponding to an average sequence coverage of about 139 times. These highly expressed CAZy genes are likely to play critical roles in the breakdown of plant fiber by rumen eukaryotes and have potential for use in cellulosic biofuel production as well as other industrial processes.

Cellulases from different families often have different substrate specificity and reaction mechanisms. Efficient degradation of the plant cell wall requires synergistic interactions between enzymes with high activity for different substrates [46]. Not surprisingly, we identified cellulases from a wide range of CAZy families. The range of glycoside hydrolases identified showed remarkable differences as compared to previous rumen metagenomic studies. For example, a large number of putative GH6, GH48, and Swollenin genes were identified. All three GH families are important for the degradation of crystalline cellulose, which is the most recalcitrant part of plant cell walls. However, these GH families were not described by earlier metagenomic approaches in the bovine rumen or wallaby foregut [10], [11]. Even in the most recent deep metagenome sequencing study [12], only three GH48 genes were identified [12], while GH6 and Swollenin were still not found. In contrast, our study identified 31 GH6, 33 GH48 and 16 Swollenin genes from rumen eukaryotes and GH6 and GH48 were among the most actively transcribed families (Table S4). These data clearly suggest that rumen eukaryotes play an important role in crystalline cellulose digestion through expression of a large amount of exo-glucanases, which were not detected in other rumen metagenomic studies.

The different CAZy families identified by rumen metagenomic studies and our metatranscriptomic study could be due not only to different nucleic acid sequencing targets, but also to the differences in the samples. Muskoxen could have developed a different plant cell wall degrading rumen microflora for survival in the arctic as compared to domesticated cows and sheep. Indeed, our preliminary analysis has identified differences in the microbial population between muskoxen and domesticated cattle (Forster, RJ, personal communication).

Assessment of the combined rumen/gut microbiome sequencing information across studies would suggest that the rumen seems to lack cellulases from GH7 and GH12 families. So far, all members of GH7, a family of exo-glucanases, have been isolated from aerobic fungi. The GH7 activity of releasing cellobiose from the reducing end of the cellulose chain may be undertaken by GH48 cellulases within the rumen. Enzymes in family GH12 have been shown to have endoglucanase and xyloglucases activities [47]. Functional aspects of these enzymes may arise from GH74 enzymes in the rumen [48].

A total of 3.4% reads showed top BLASTX matches to bacteria (Figure 2) and over half of these reads exhibited the highest similarity to proteins from bacteria that are known as predominant rumen/gut residents, such as families of *Fibrobacteraceae*, *Clostridiaceae*, *Ruminococcaceae*, *Prevotellaceae*, *Bacteroidaceae*, and *Lachnospiraceae* (Figure S6). However, these bacteria-like reads are very unlikely to come from bacterial mRNA because only enriched polyA RNAs were sequenced, which were rarely found in bacteria mature mRNA. Phylogenetic binning using protein markers also confirmed the absence of bacteria-derived genes in our dataset [Figure S7]. In addition, the GC content of the “bacteria-like” genes identified from muskoxen transcriptome were about 40.1%, which is also within the range of rumen eukaryotic genes identified, but much lower than the average GC content of the metagenomic studies which represents the bacterial population (Figure S2). These findings imply that bacteria-like genes, most of which are involved in a variety of metabolic functions (Figure S8) (including 35% of all CAZy genes identified), may have been horizontally transferred into the genome of rumen eukaryotes, most likely from rumen bacteria, which was demonstrated before [49], [50].

Anaerobic fungi and protozoa are the two major groups of eukaryotes in the rumen [51], [52]. In the present study, functional based phylogenetic binning (Figure 2), top BLAST matches of the CAZy enzymes (Figure S9) and phylogenetic analysis based on SSU ribosome RNA sequence (data not shown), all indicated the presence of both groups. There were significantly (χ^2^ = 348, p<0.0001) more CAZy enzymes matching to rumen fungi than to rumen protozoa (Figure S9), indicating that rumen fungi may play a more significant role for fiber digestion in the muskoxen rumen. However, since there are more CAZy genes from rumen fungi than from protozoa in the nr database (101 vs 28, Table S4), the differences could be due in part to the number of homologues currently present in the database.

Eukaryotic anaerobic microbes are poorly understood, especially from a molecular perspective. Although this study focused primarily on genes encoding for enzymes involved in plant cell wall degradation, the data presented greatly expands our current knowledge of these unique eukaryotes and should provide further insight into their co-evolution, metabolism and function within the rumen microbial community.

## Materials and Methods

### Ethics statement

The animals were cared for according to procedures that were approved under protocol # 139821-2 by the Institutional Animal Care and Use Committee at the University of Alaska Fairbanks, Fairbanks, AK.

### Muskoxen rumen sampling

Ruminal samples were obtained from two cannulated muskoxen at the University of Alaska Fairbanks (Fairbanks, AK) in September, 2009. The muskoxen were mature male castrates with mean body mass between 245 and 271 kg. During a 1-month period, the muskoxen were fed a triticale (*Triticosecale*) straw or a brome grass hay (*Bromus* sp.) based high fiber diet, offered twice daily, plus a small amount of protein and mineral supplement once in the morning (335 g/d). See supporting Table S1 for the feed composition. During the last week of the period, rumen samples were obtained in the morning, before the muskoxen were fed. The ruminal contents were transferred to a heavy walled 250 ml beaker and the solid and liquid phases were separated using a Bodum coffee filter plunger (Bodum Inc., Triengen, Switzerland). Subsamples of solid digests (∼2.5 g) were immediately flash-frozen in liquid nitrogen. All samples were frozen within 5 min of the sample being withdrawn from the animal. Samples were immediately transferred to the lab, and stored at −80°C until further processing.

### RNA extraction and purification

Total RNA was isolated from rumen solids according to Wang et al. [37]. The quality of total RNA was estimated by running the samples on RNA 6000 nano chip on an Agilent 2100 BioAnalyzer.

### RNA sequencing and sequence assembly

mRNA-Seq libraries were constructed from 100 µg of total RNA using the Illumina mRNA-Seq sample preparation kit according to the manufacturer's instructions (Illumina Inc, San Diego, USA). Two samples from two individual muskoxen (one fed triticale straw, one fed brome grass hay) were combined and sequenced using the Illumina Genome Analyze II system at the McGill University/Genome Quebec Innovation Centre. Obtained sequencing reads were assembled *de novo* using a combination of Velvet (available at http://www.ebi.ac.uk/~zerbino/velvet/) [53] and CAP3 (available at http://pbil.univ-lyon1.fr/cap3.php) [54] programs as described in Methods S1 and validated by reverse transcription PCR. The contigs were then clustered together at 95% sequence identity over 80% of their lengths using the DNA version of CD-HIT in the Rammcap package [55].

To validate the contig assemblies, 20 contigs containing different putative carbohydrate active enzyme genes were amplified using primers designed specifically for each contig. The target contigs and primer sequences are listed in Table S2. Briefly, the muskoxen total RNA samples were reverse transcribed using a Superscript III kit and oligo-dT primers (Invitrogen). PCRs were carried out using Platinum *Taq* polymerase Hi Fidelity (Invitrogen) using the conditions recommended by the manufacturer.

### Sequence analysis

All sequence analyses, unless otherwise specified were performed using both the reads and the assembled contigs. The databases employed for this analysis were the latest versions available during the analysis period (Jun 2010 to Dec 2010). The genome sequence of the rumen bacterium *Fibrobacter succinogenes* S85 (Accession No. NC_013410), expressed sequence tags (ESTs) of rumen protozoa [50] and rumen fungi *Pyromyces* sp. E2, were retrieved from National Center for Biotechnology Information (NCBI) Genbank databases (http://www.ncbi.nlm.nih.gov/).

### rRNA sequence identification

Ribosomal RNA sequences were firstly identified by BLASTN searches against LSU and SSU ribosomal RNA databases (Version 104) from the ARB-Silva database [56]. Subsequently, all the sequences were further analyzed by the rRNA-hmm [57] and tRNA-scan [58] programs in the Rammcap package [55], using the default settings. The sequences that had E-values equal or less than 10^−5^ (bit score ≥52) and overlap ≥50 bp to entries in the SSU/LSU database, as well as those identified by rRNA-hmm program (SSU rRNA, LSU rRNA and tRNA) are referred to as non-coding RNA (ncRNA) in this paper.

### Binning

Function-based taxonomy binning was carried out with the top BLASTX hits to the trimmed down non-redundant amino acid database (NRMO) using the MEGAN software [59] as well as phylogenetic analysis on protein coding marker gene sequences (Methods S1).

### Functional annotation of the non-ncRNA sequences

The non-ncRNA sequences were searched using RPS-BLAST against both the KOG and the COG databases and the Genbank non-redundant amino acid (nr) database. Bacteria-like reads identified by nr BLASTX were further searched against the COG database. The functional roles of the sequences were assigned based on the KOG and COG searches. The matches that had E-values equal or less than 10^−5^ were considered significant.

### Carbohydrate active protein annotation

Lignocellulolytic gene containing reads and contigs were identified and classified based on the Carbohydrate Active enZYme (CAZy) database [60] as described by Warnecke and colleagues [13] with the following modification. Both HMMER3 [61] and BLASTX searches were carried out as follows: Step A) Glycoside hydrolase and carbohydrate binding module (CBM) families that have associated Pfam HMMs (v24.0) [11] were used directly for HMMER hmmsearch. Step B) In an attempt to associate Pfam HMMs to CAZy families without such models, all members of these CAZy families were searched against the Pfam-A and Pfam-B databases (v24.0). Results were manually checked and Pfam HMMs were conservatively chosen for a CAZy family only when the following two criteria were met: i) all hits to that Pfam group were from the same CAZy family; ii) At least 80% of group members were identified to conform to the conserved Pfam model. In instances where one Pfam HMM model represented members from two or more closely related CAZy groups, a class of combined CAZy groups was assigned. Step C) For those CAZy families that currently are not represented by a Pfam model, the representative sequences as described by Warnecke et al [13], were used in BLASTX searches with a score cut-off of 52. Step D) For CAZy families with neither a Pfam accession nor representative amino acid sequences, an HMM profile was built based on T-coffee [62] alignment of representative members selected from the CAZy web site and used for HMMER3.

### Glycoside hydrolase cluster analysis

To compare genes coding for carbohydrate active proteins identified in the muskoxen rumen metatranscriptome with other genomes/metagenomes, the percentages of glycoside hydrolase families were calculated. A two-dimensional matrix was constructed, consisting of the GHs that were identified from genomes or metagenomes, wherein each cell in the matrix indicated how often a GH family was seen within a particular sample. Pearson correlation coefficients of each two samples were calculated and transformed into distances and clustered by using the unweighted pair group method with the arithmetic mean algorithm as previously described [49], [63].

### Sequence Data Availability

The sequencing reads are available from the NCBI short read archive under Accession number SRA030623.1.

## Supporting Information

Methods S1 **Short read assembly and analysis.**
(DOC)

Figure S1 **Length distribution of muskoxen rumen metatranscriptome contigs.** The number of contigs is indicated on the right side of the bar.
(TIF)

Figure S2 **GC content analysis of the muskox rumen microbial community metatranscriptome.** The % GC of each contig was calculated. Number shown on the column indicating number of contigs with a certain GC range. The data of ocean microbiome metatranscriptome, bovine rumen metagenome and termite gut metagenome are also shown.
(TIF)

Figure S3 **Top 30 phylogenetic bins of the muskoxen rumen metatranscriptome as determined by comparison against NCBI's non-redundant protein database (nr).** Ranks are determined by the highest total reads number at the genus level.
(TIF)

Figure S4 **Top 30 KOG bins of the muskoxen rumen metatranscriptome as determined by comparison against KOG database.** Ranks are determined by the highest number of total reads for each KOG category.
(TIF)

Figure S5 **Schematic representation of plant cell wall polysaccharide and energy metabolism of the muskoxen rumen eukaryotic population.** The inner box represents the hydrogenosome present in anaerobic fungi and possibly the rumen protozoa. The number after each enzyme represents the read number identified by KOG/COG searches. Abbreviations: ASCT, Acetate: Succinate CoA-transferase; CAZY, carbohydrate active enzymes; GAPDH, glyceraldehydes-3-phosphate dehydrogenase; PEP, phosphoenolpyruvate; PFL, Pyruvate: Formate lyase; PFO, Pyruvate: ferredoxin oxidoreductase; PGK, Phosphoglycerate kinase; PGM, Phosphoglycerate mutase.
(TIF)

Figure S6 **Top 30 phylogenetic bins of the bacterial reads of muskoxen rumen metatranscriptome as determined by comparison against NCBI's non-redundant protein database (nr).** Ranks are determined by the highest number of total reads at the family level.
(TIF)

Figure S7 **Evidence of eukaryotic origin of the metatranscriptome sequences based on BLASTX searches of a) reads that were assigned to actin (KOG0676) b) reads that were assigned to translation elongation factor EF1 (KOG0052) and c) MLTreeMap analysis of all the contigs.** Number of reads that matched to each node are indicated in a) and b). Please refer to supplementary methods for details.
(TIF)

Figure S8 **Gene category distribution of the muskoxen rumen metatranscriptome as annotated using Eukaryotic Orthologous Groups (KOGs, for reads showing top BLASTX match to eukaryotic genes; Blue color) and clusters of orthologous groups (COGs, reads showing top BLASTX match to bacterial genes; Orange color).** A total of 5.7 million out of 21.1 million putative protein encoding sequences in the muskoxen rumen eukaryotic metatranscriptome were annotated to a KOG category or COG category. The percentage of annotated ORFs for each KOG/COG category is shown.
(TIF)

Figure S9 **Phylogenetic distribution of muskoxen rumen metatranscriptome putative carbohydrate active enzymes based on MEGAN analysis of top BLASTX hits of the contigs against the Genbank non-redundant amino acid database.** The number of contigs (≥500 bp) that matched to each node is indicated.
(TIF)

Table S1 **Feed composition.**
(DOC)

Table S2 **Primers used for validating lignocellulolytic enzyme related contigs.**
(DOC)

Table S3 **Metabolic related KOG/COG groups represented by 5000 or more reads in the metatranscriptomes from Muskoxen rumen eukaryotes.**
(DOC)

Table S4 **The abundance of contigs coding lignocellulytic enzymes [glycoside hydrolases (GHs), carbohydrate esterases (CEs), pectate lyases (PLs), carbohydrate-binding modules (CBMs), and other related modules] in the muskoxen eukaryotic metatranscriptome (Muskoxen MT) and a comparison of their abundance in our databases of rumen fungal (Ru. Fungi) and rumen protozoal genes (Ru. prot.) as well as different anaerobic bacteria, including** ***Bacteroides fragilis*** **(Bfra),** ***Butyrivibrio proteoclasticus*** **(Bpro),** ***Clostridium thermocellum*** **(Cthe),** ***Fibrobacter succinogenes*** **(Fsuc),** ***Prevotella ruminicola*** **(Prum),** ***Ruminococcus flavifaciens*** **(Rfla), and the rumen fungus** ***Piromyces*** **sp. E2 ESTs (Pir. ESTs).**
(DOC)

Table S5 **Muskoxen rumen metatranscriptome contigs (≥500 bp) that have one known CAZY module.**
(XLS)

Table S6 **Muskoxen rumen metatranscriptome contigs (≥500 bp) that have 2 or more distinct CAZY modules.**
(XLS)
