# Supplementary material for: Snapshot of the Eukaryotic Gene Expression in Muskoxen Rumen—A Metatranscriptomic Approach
Source: PLoS One. 2011 May 31;6(5):e20521. doi: 10.1371/journal.pone.0020521 (PMC3105075; doi:10.1371/journal.pone.0020521)
Supplement: Methods S1 — Short read assembly and analysis. (DOC) [file pone.0020521.s001.doc]

**Methods S1.** Short reads assembly and analysis.

*Short read transcript assembly*

The initial RNA-seq data set consisted of 26061208 108-mer reads in fastq format. The data set was split into 13 separate data sets of 2000000 reads each (plus a remaining one with 244832 reads). On each of these split data sets, the program Velvet suite of programs (52) was run. Velvet was run with three different k-mer parameters; k=37, k=45 and k=53. For each of these, the velveth and velvetg programs were run.

Following this step for all 14 split data sets the resulting contigs in the directories were concatenated into a single contigs file. The velveth and velvetg programs were run again on this dataset, which is effectively a set of long reads of varying length in fasta format, also with k=37

The steps above were repeated for k=45 and k=53. The three final “contigs.fa” files for these 3 k-mer parameters were then concatenated into a single contigs file.. The program CAP3 was then run on this file, with default parameters. The files with extensions .contigs and .singlets were concatenated into a single file which represented the assembled transcript contigs in the present study. This method of transcript assembly was selected following extensive experimentation and produces more long contigs containing full length transcripts when compared with traditional assembly methods which are more suited to DNA reads.

*Analysis*

The functional based taxonomic assignment was constructed by the Metagenomic Analyzer (MEGAN) software [1] based upon the best BLASTX hit to an in-house database named as NRMO. The NRMO database contained all protein sequences in the Genbank non redundant database (nr) that had a match to any of our assembled contigs, with an e-value no greater than 1.0E-5. There were about 230, 000 entries in the NRMO database. To validate the NRMO database, 20000 reads were randomly picked and BLASTed against both the nr database and NRMO database respectively, and compared. The results, especially for the taxonomy distribution at genus level, were very similar to each other (data not shown). Collector’s curves were produced from an *ad hoc* Perl script and plotted in Microsoft Excel version 2003. The taxonomic composition was also estimated by running the software MLTreeMap on the assembled contigs as described [2]. Putative full-length genes were identified as follows: The assembled contigs were BLASTX-ed against the UniProt database. Contigs were then translated in the proper reading frame based on the BLASTX hits. The resulting amino acid sequences were searched for all full length ORFs of at least 70 amino acids which fully encompass the alignment of the BLAST Hits.

1. Huson, D.H., et al., *MEGAN analysis of metagenomic data.* Genome research, 2007. **17**(3): p. 377-86.

2. Stark, M., et al., *MLTreeMap--accurate Maximum Likelihood placement of environmental DNA sequences into taxonomic and functional reference phylogenies.* BMC genomics, 2010. **11**: p. 461.
