# Supplementary figures and images for: Snapshot of the Eukaryotic Gene Expression in Muskoxen Rumen—A Metatranscriptomic Approach
Source: PLoS One. 2011 May 31;6(5):e20521. doi: 10.1371/journal.pone.0020521 (PMC3105075; doi:10.1371/journal.pone.0020521)

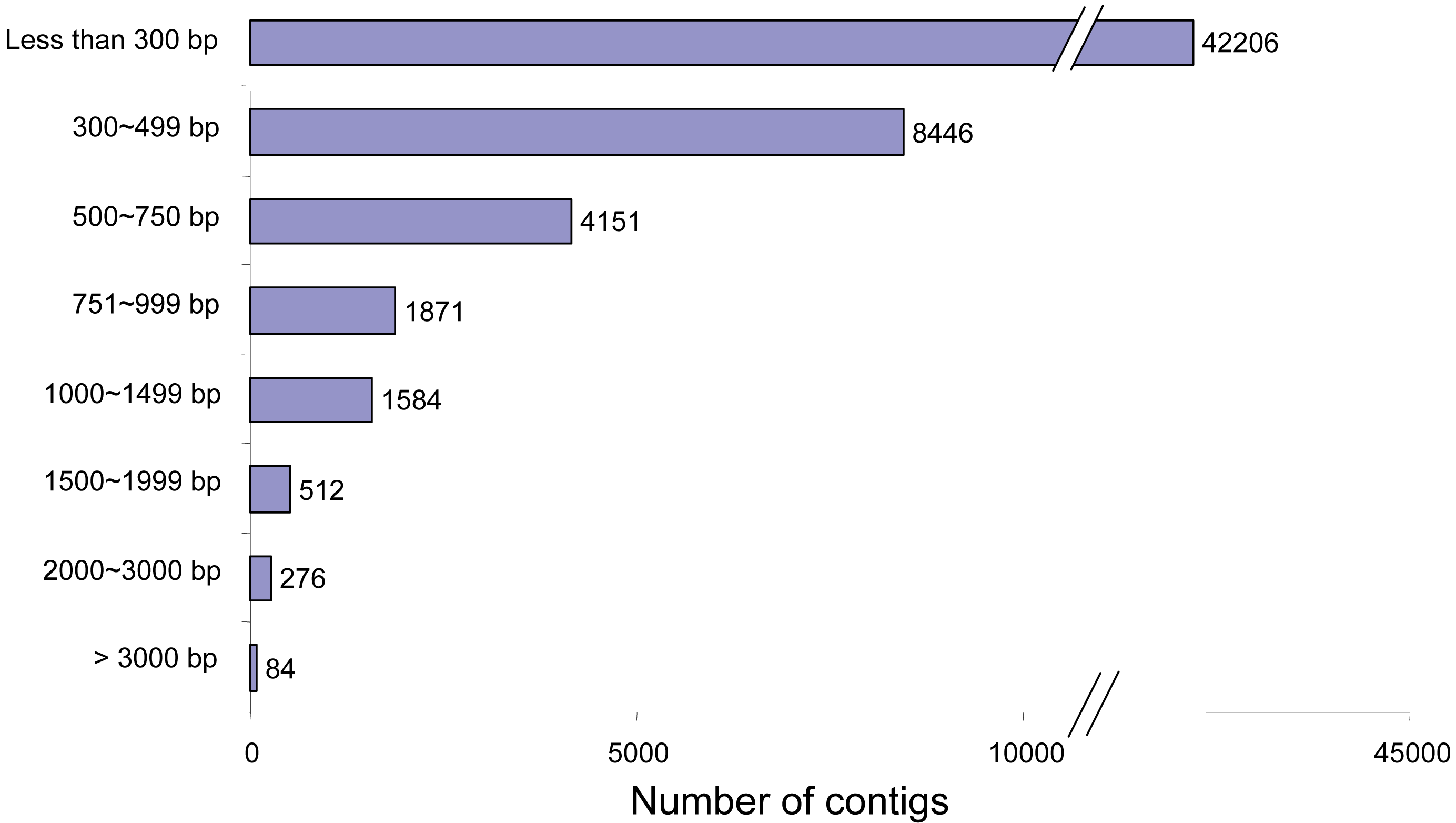

Supplement: Figure S1 — Length distribution of muskoxen rumen metatranscriptome contigs. The number of contigs is indicated on the right side of the bar. (TIF) [file pone.0020521.s002.tif]

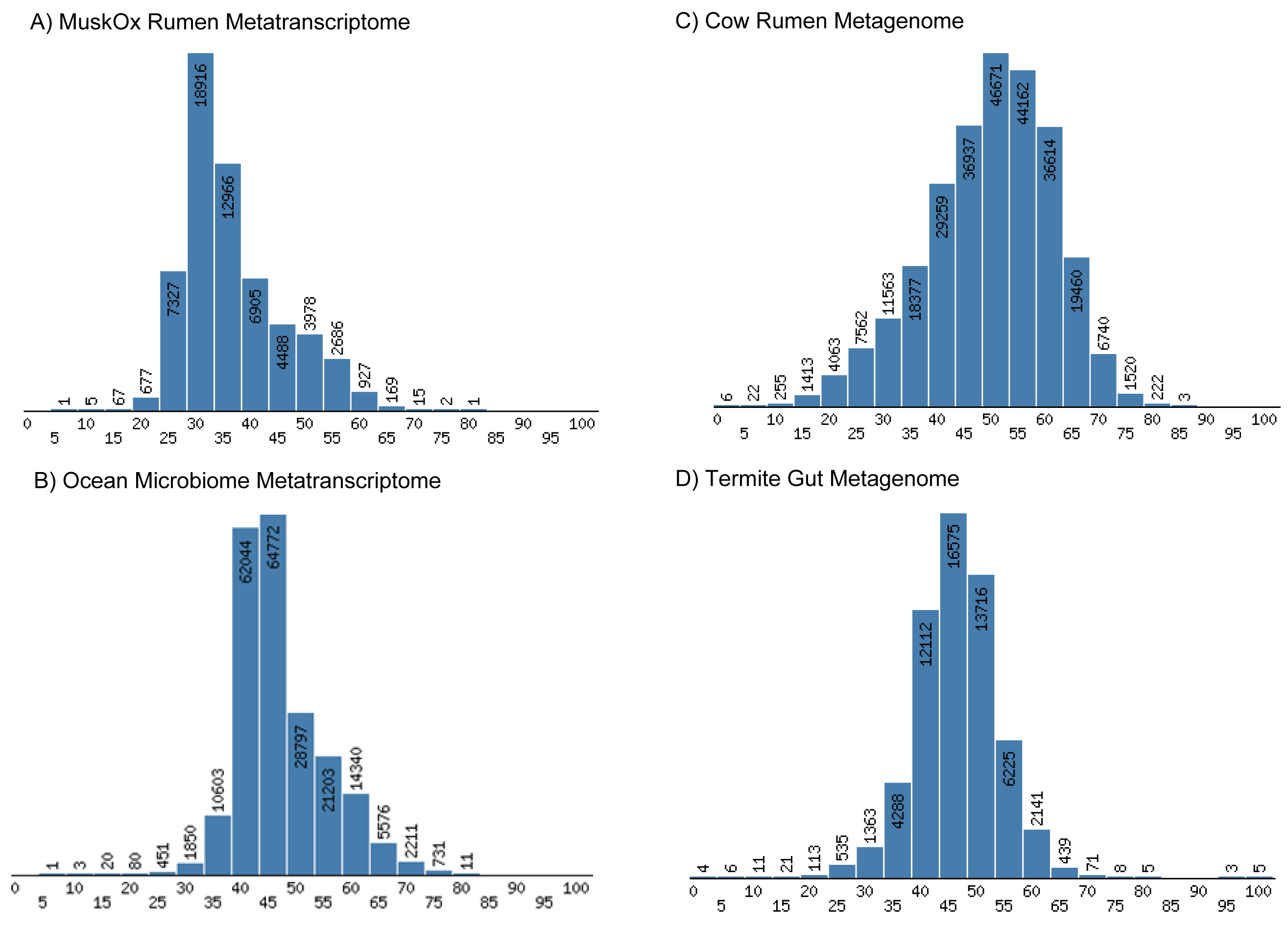

Supplement: Figure S2 — GC content analysis of the muskox rumen microbial community metatranscriptome. The % GC of each contig was calculated. Number shown on the column indicating number of contigs with a certain GC range. The data of ocean microbiome metatranscriptome, bovine rumen metagenome and termite gut metagenome are also shown. (TIF) [file pone.0020521.s003.tif]

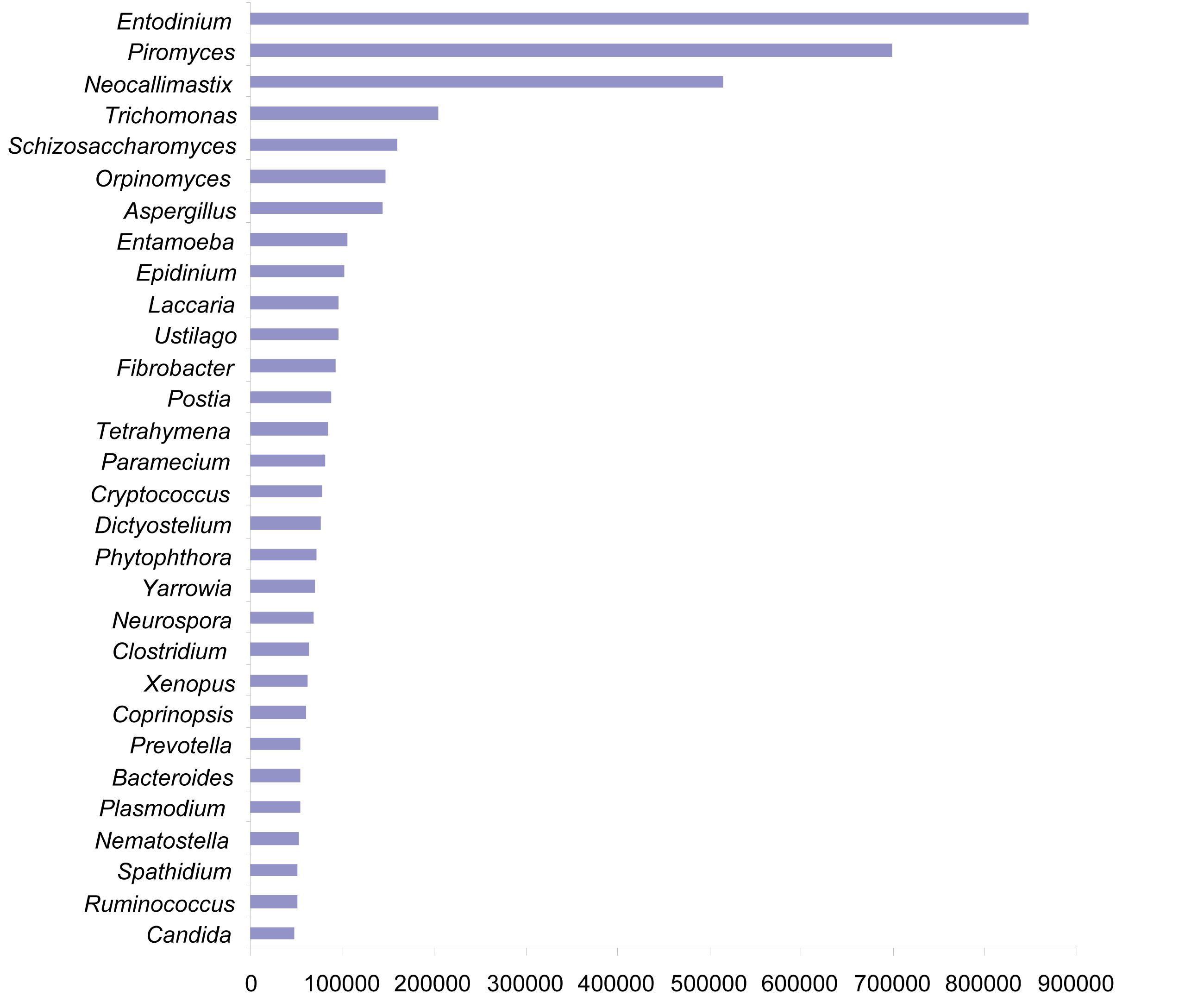

Supplement: Figure S3 — Top 30 phylogenetic bins of the muskoxen rumen metatranscriptome as determined by comparison against NCBI's non-redundant protein database (nr). Ranks are determined by the highest total reads number at the genus level. (TIF) [file pone.0020521.s004.tif]

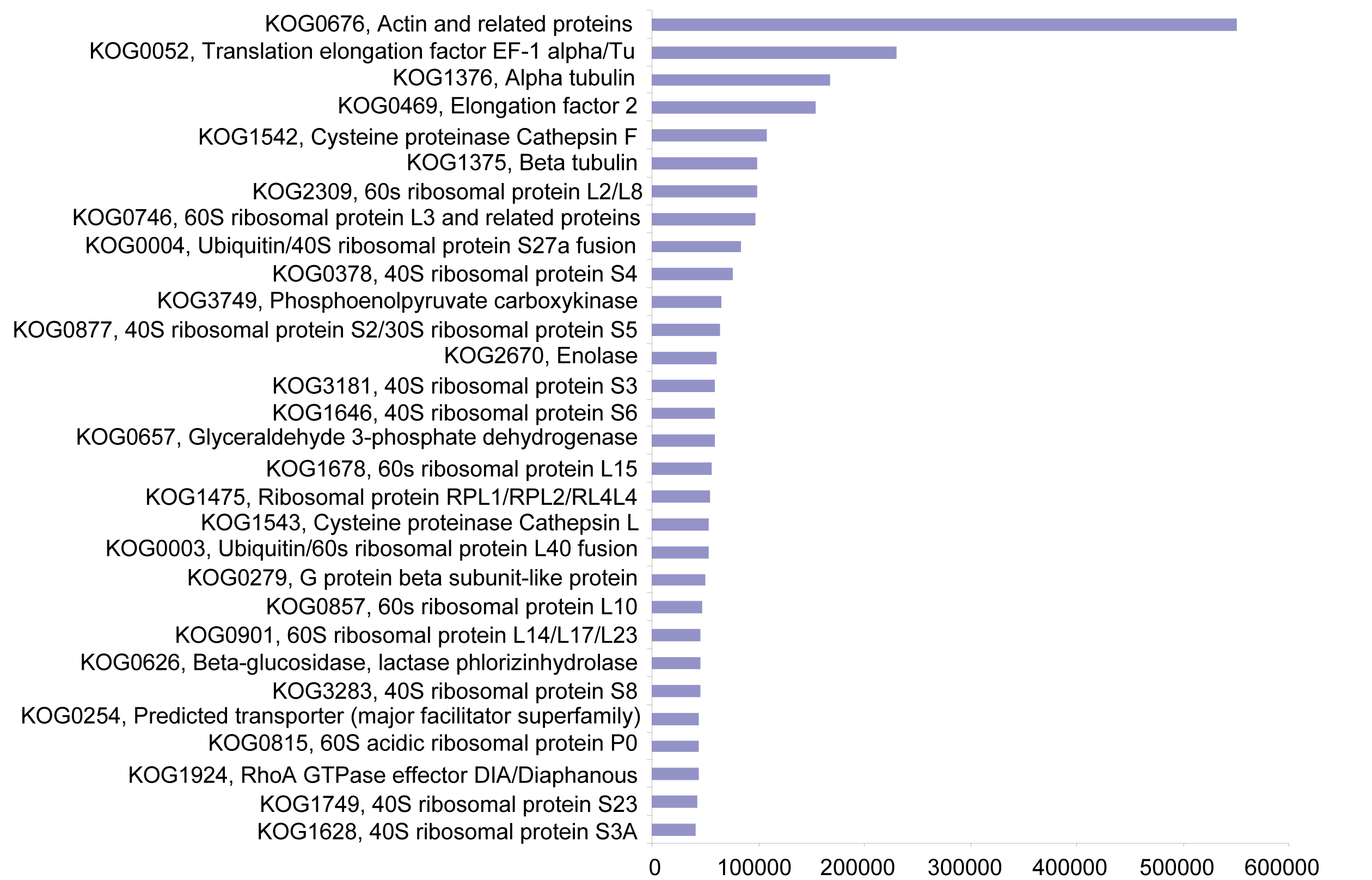

Supplement: Figure S4 — Top 30 KOG bins of the muskoxen rumen metatranscriptome as determined by comparison against KOG database. Ranks are determined by the highest number of total reads for each KOG category. (TIF) [file pone.0020521.s005.tif]

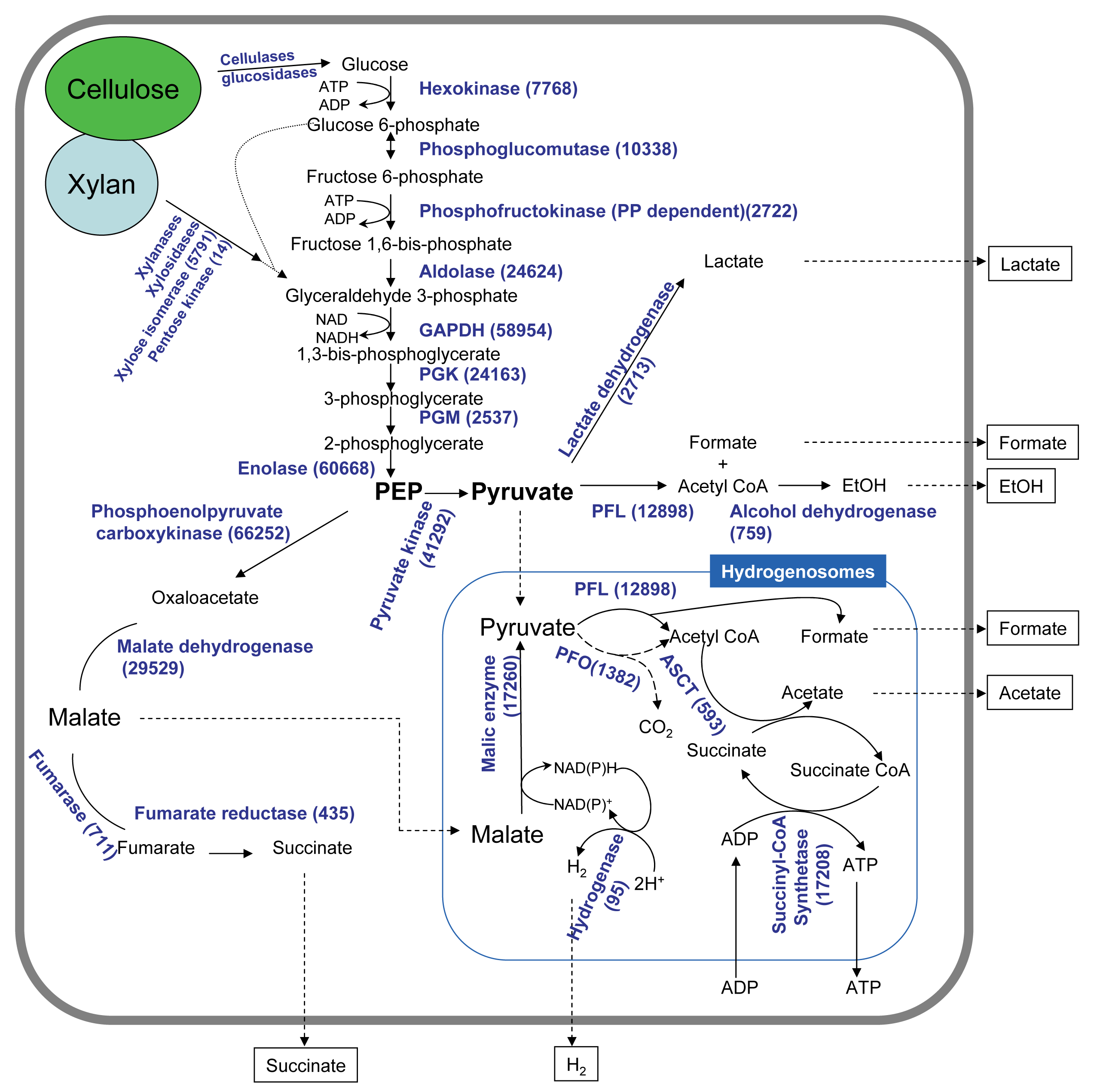

Supplement: Figure S5 — Schematic representation of plant cell wall polysaccharide and energy metabolism of the muskoxen rumen eukaryotic population. The inner box represents the hydrogenosome present in anaerobic fungi and possibly the rumen protozoa. The number after each enzyme represents the read number identified by KOG/COG searches. Abbreviations: ASCT, Acetate: Succinate CoA-transferase; CAZY, carbohydrate active enzymes; GAPDH, glyceraldehydes-3-phosphate dehydrogenase; PEP, phosphoenolpyruvate; PFL, Pyruvate: Formate lyase; PFO, Pyruvate: ferredoxin oxidoreductase; PGK, Phosphoglycerate kinase; PGM, Phosphoglycerate mutase. (TIF) [file pone.0020521.s006.tif]

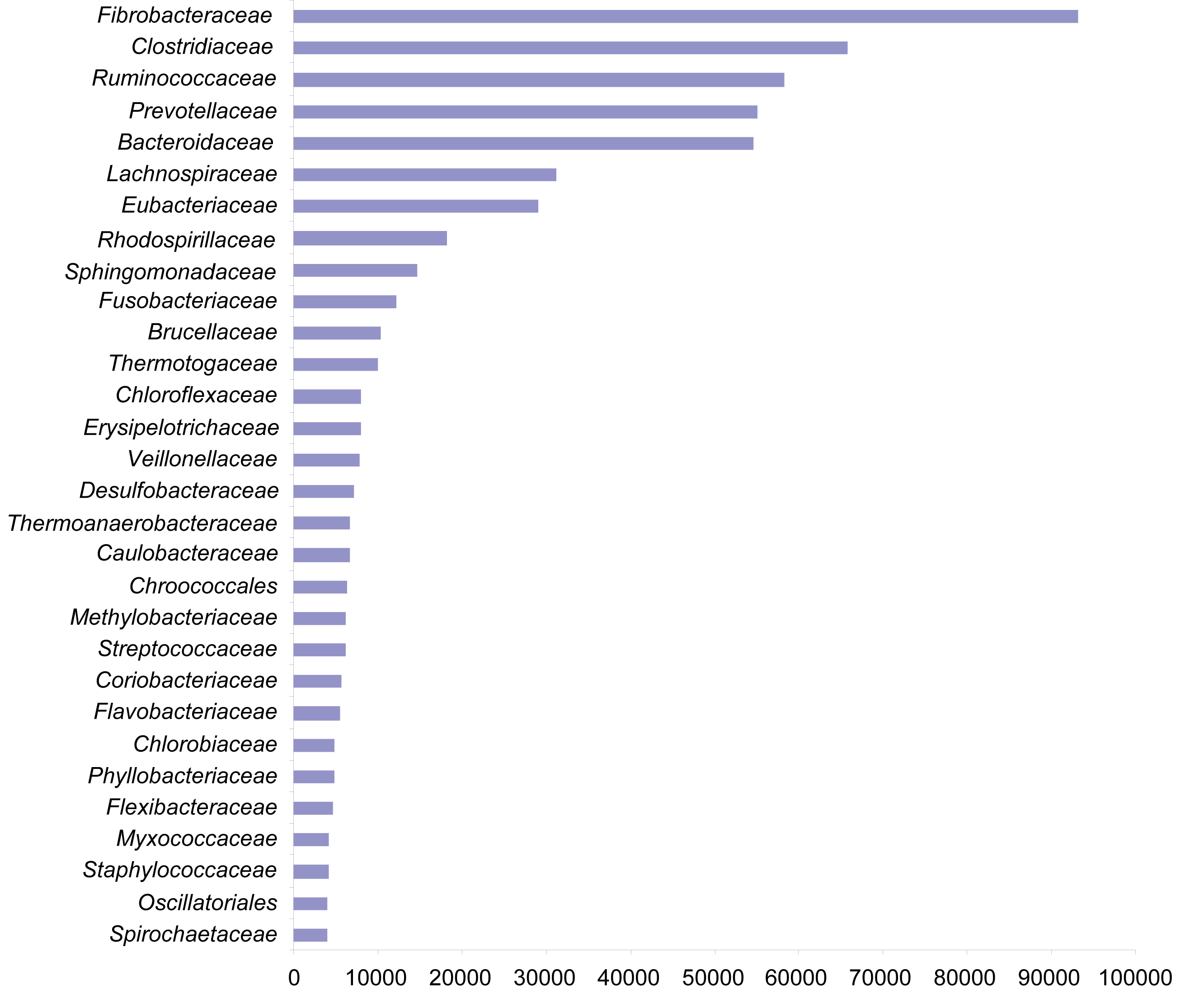

Supplement: Figure S6 — Top 30 phylogenetic bins of the bacterial reads of muskoxen rumen metatranscriptome as determined by comparison against NCBI's non-redundant protein database (nr). Ranks are determined by the highest number of total reads at the family level. (TIF) [file pone.0020521.s007.tif]

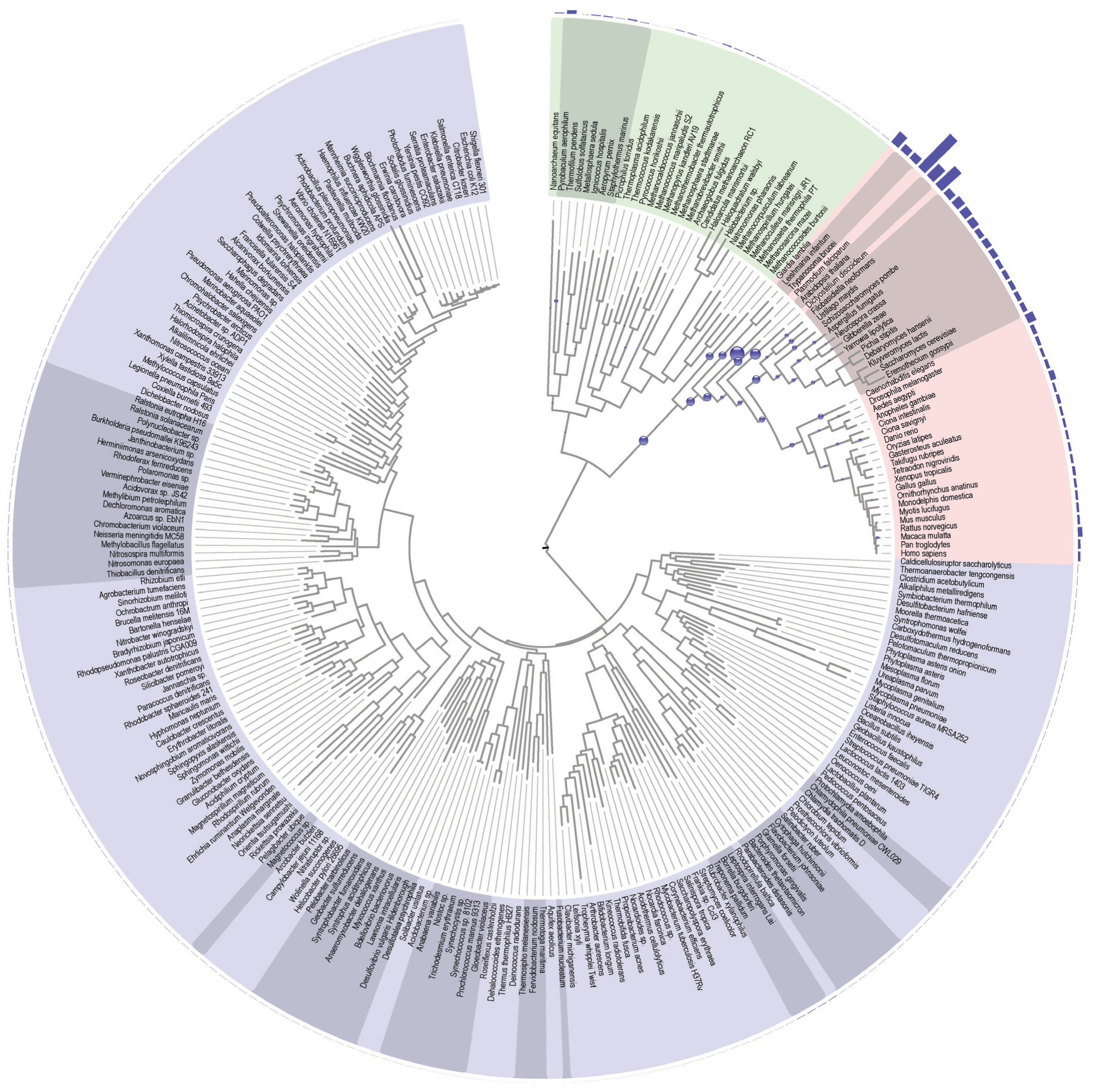

Supplement: Figure S7 — Evidence of eukaryotic origin of the metatranscriptome sequences based on BLASTX searches of a) reads that were assigned to actin (KOG0676) b) reads that were assigned to translation elongation factor EF1 (KOG0052) and c) MLTreeMap analysis of all the contigs. Number of reads that matched to each node are indicated in a) and b). Please refer to supplementary methods for details. (TIF) [file pone.0020521.s008.tif]

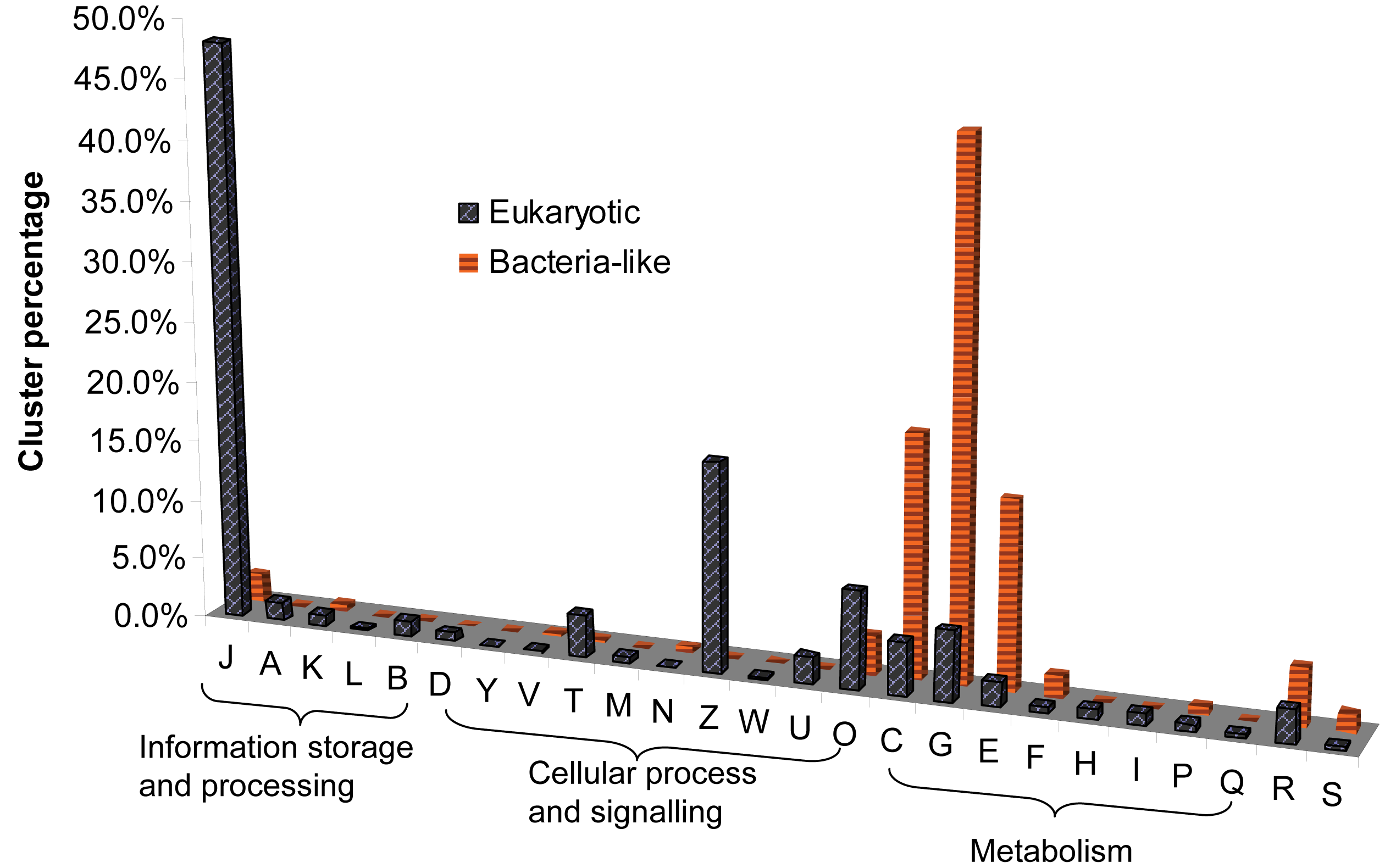

Supplement: Figure S8 — Gene category distribution of the muskoxen rumen metatranscriptome as annotated using Eukaryotic Orthologous Groups (KOGs, for reads showing top BLASTX match to eukaryotic genes; Blue color) and clusters of orthologous groups (COGs, reads showing top BLASTX match to bacterial genes; Orange color). A total of 5.7 million out of 21.1 million putative protein encoding sequences in the muskoxen rumen eukaryotic metatranscriptome were annotated to a KOG category or COG category. The percentage of annotated ORFs for each KOG/COG category is shown. (TIF) [file pone.0020521.s009.tif]

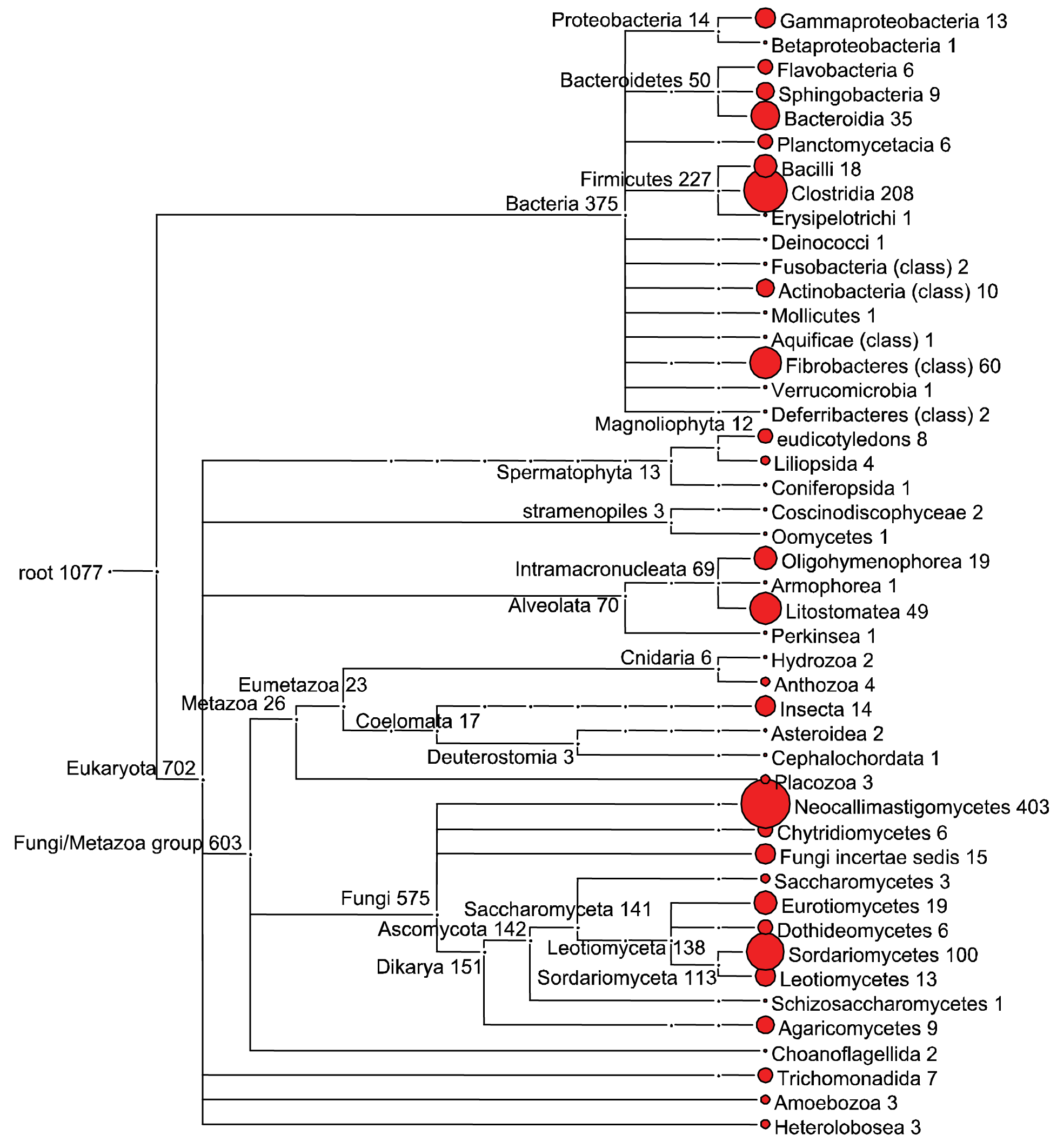

Supplement: Figure S9 — Phylogenetic distribution of muskoxen rumen metatranscriptome putative carbohydrate active enzymes based on MEGAN analysis of top BLASTX hits of the contigs against the Genbank non-redundant amino acid database. The number of contigs (≥500 bp) that matched to each node is indicated. (TIF) [file pone.0020521.s010.tif]
