# Supplementary material for: Snapshot of the Eukaryotic Gene Expression in Muskoxen Rumen—A Metatranscriptomic Approach
Source: PLoS One. 2011 May 31;6(5):e20521. doi: 10.1371/journal.pone.0020521 (PMC3105075; doi:10.1371/journal.pone.0020521)
Supplement: Table S1 — Feed composition. (DOC) [file pone.0020521.s011.doc]

**Table S1** Feed composition

| Feed | DM (g/g) | NDF  (g/g DM) | ADF  (g/g DM) | Cellulose (g/g DM) | Hemicellulose (g/g DM) | Lignin (g/g DM) | N  (g/g DM) |
| --- | --- | --- | --- | --- | --- | --- | --- |
| Hay | 0.875 | 0.699 | 0.378 | 0.308 | 0.321 | 0.066 | 0.0112 |
| Straw | 0.878 | 0.837 | 0.522 | 0.437 | 0.315 | 0.074 | 0.00673 |
| SEM | 1.82 × 10^-3^ | 0.0143 | 0.0150 | 0.0175 | 2.40 × 10^-3^ | 0.0135 | 0.0464 |
| P = | 0.49 | 0.02 | 0.02 | 0.007 | 0.19 | 0.71 | < 0.001 |
