# Supplementary material for: Snapshot of the Eukaryotic Gene Expression in Muskoxen Rumen—A Metatranscriptomic Approach
Source: PLoS One. 2011 May 31;6(5):e20521. doi: 10.1371/journal.pone.0020521 (PMC3105075; doi:10.1371/journal.pone.0020521)
Supplement: Table S2 — Primers used for validating lignocellulolytic enzyme related contigs. (DOC) [file pone.0020521.s012.doc]

**Table S2.** Primers used for validating lignocellulolytic enzyme related contigs

| **Primer Name** | **Primer sequence** | **Target Contig** | **Contig Length** | **Position** | **Theoretical Product Size** | **CAZY Family** |
| --- | --- | --- | --- | --- | --- | --- |
| 30088_62_outerF | ATGGTGGTGATAACAACTCTGG | Contig30088 | 1072 | 62 | 941 | CE1 |
| 30088_70_outerR | CCCATTCTACCGTCACCTTC |  |  | 1003 |  |  |
| 30088_265_innerF | AGTCTTAAGAGTAACACCACCC |  |  | 265 | 423 |  |
| 30088_385_innerR | AGATCAAAGGCTGATGGAGCAG | |  | 688 |  |  |
| 29149_62_outerF | TTACCATTACCTTCACCGTGACCTC | Contig29149 | 1132 | 62 | 967 | CE1 |
| 29149_104_outerR | TTTCCCAGGCGGCGGTATGG |  |  | 1029 |  |  |
| 29149_200_innerF | GCCCATTCAGCTAAGTTACCC |  |  | 200 | 718 |  |
| 29149_215_innerR | GGGTGGATTCACTCAAGATGA |  |  | 918 |  |  |
| 2424_106_outerF | GAGCACCAACACAAGCACTAG | Contig2424 | 1033 | 106 | 832 | CE3 |
| 2424_96_outerR | GTGGTATGGGTGGTATGTTCG |  |  | 938 |  |  |
| 2424_280_innerF | CTTGATCAGTACCCATATCAGC |  |  | 280 |  |  |
| 111_106_outerF | GGGTTGTACAGTTGAATACACCG | Contig111 | 1476 | 106 | 1255 | GH6 |
| 111_116_outerR | CCAGCATCTGGAGCACCTTG |  |  | 1361 |  |  |
| 111_315_innerF | GTGGTATTCCAAGCAAATGTGG |  |  | 315 | 856 |  |
| 111_300_innerR | ACCAGGTACCAGAAGCGTTG |  |  | 1171 |  |  |
| 22047_114_outerF | GGCATGGATAGCACAAAGATTG | Contig22047 | 1921 | 114 | 1694 | GH6 |
| 22047_114_outerR | TGGAGCTGGTTTCATGGCAG |  |  | 1808 |  |  |
| 22047_450_innerF | TGCTCTTGCCGCTAAGGTCTC |  |  | 450 | 1172 |  |
| 22047_300_innerR | ACCTGGGTGCTTACGGTCAG |  |  | 1622 |  |  |
| 30327-o1 | GGAAATGGTTCTTGGGGTGTAG | Contig30327 | 1485 | 98 | 1252 | CE1 |
| 30327-outer2C | CCTGGAGTGTTTGCTTTTGG |  |  | 1350 |  |  |
| 30327-I1 | GGGGTCAAAACAACCAAG |  |  | 420 | 651 |  |
| 30327-I2c | GACATACCACCACCCATT |  |  | 1071 |  |  |
| 30515-o1 | GACAGCATGGGTAACCTA | Contig30515 | 1129 | 93 | 911 | CE6 |
| 30515-o2c | CTGCTATTCCACCACTTG |  |  | 1004 |  |  |
| 30515-I1 | TTCGGCGTCAGTAGTATCTTCC |  |  | 391 | 503 |  |
| 30515-I2c | GCCGTTCCAGGTTGTGATATTC |  |  | 894 |  |  |
| 29571-O1 | CTGTCCCACCACTTGCTAATTG | Contig29571 | 1198 | 233 | 912 | CE6 |
| 29571-O2c | TCGACATCCCACTTACCATCAG |  |  | 1145 |  |  |
| 29571-I1 | TGTTGCTGTTGCTGGTTGTG |  |  | 339 | 745 |  |
| 29571-I2c | AACATGGGTAGCCAAGATCC |  |  | 1084 |  |  |
| 1733-o1 | CCAAAACCACCACCTGGAAT | Contig1733 | 1399 | 256 | 1018 | GH45 |
| 1733-o2c | GGTGAATGGGGTGTTGAAAACG | |  | 1274 |  |  |
| 1733-I1 | AGCAGCAGCGTAACCATAGGA |  |  | 420 | 538 |  |
| 1733-I2c | CGGATTGTGTTGCAGCTTGGA |  |  | 958 |  |  |
| 32-O1 | GGGCAACAAGCCAAGGTTAC | Contig32 | 1284 | 113 | 998 | GH45 |
| 32-O2c | GCTTTAAGGCAGCTGGAAGG |  |  | 1111 |  |  |
| 32-I1 | TGATGGTAAGTGGGCCATTG |  |  | 315 | 635 |  |
| 32-I2c | CCACCAGTGTTGGTTGTTTG |  |  | 950 |  |  |
| 29098-O1 | GGCTGGTAAGGTCTGTAGAG | Contig29098 | 1484 | 71 | 1228 | GH48 |
| 29098-O2c | CCATGTCACCACCGAAAC |  |  | 1299 |  |  |
| 29098-I1 | GGGACAAGTTGAAGACTACC |  |  | 539 | 313 |  |
| 29098-I2c | TGTTGGATGGAGCCTTAC |  |  | 852 |  |  |
| Node3576-O1 | TCCATCTTCACCAGCTACCTATGC | Contig_Node3576 | 2067 | 123 | 1534 | GH48 |
| NODE3576-O2c | CCCAGAATCTGTGGTAACGGAATC | |  | 1657 |  |  |
| NODE3576-I1 | GCTCGTGCTATTCAAGGTGCTTAC | |  | 508 | 718 |  |
| NODE3576-I2c | CTACCGTCCCAAGAAAGGTTTGTG | |  | 1226 |  |  |
| 2118-I1 | TGGGTCTTGGTTGCATAC | Contig2118 | 2396 | 866 | 1026 | GH74 |
| 2118-I2c | TGCTGGAGGTGATGTTAC |  |  | 1892 |  |  |
| 2118-O1 | TTTCTGGGGCTCCATATC |  |  | 295 | 1959 |  |
| 2118-O2c | GTCTCGGGTATTGTTGTC |  |  | 2254 |  |  |
| 24305-O1 | CAAATGGCCTACATGGACTGACC | Contig24305 | 1399 | 262 | 1012 | GH74 |
| 24305-O2c | CACCTGTACCACCTGCTTCTTTC |  |  | 1274 |  |  |
| 24305-I1 | TTGCTGGATTGGCCTTCGGAGGAT | |  | 516 | 436 |  |
| 24305-I2C | GGCGAAAACACCATTTCCTG |  |  | 952 |  |  |
| 23421-O1 | CTCCAGCTATTGCCCAATTCG | Contig23421 | 1355 | 65 | 1127 | CE3 |
| 23421-O2C | CAGCAGCTCCATTACCACAAC |  |  | 1192 |  |  |
| 23421-I1 | GGTATGTTCGGTGGAGGTCAAAG | |  | 235 | 824 |  |
| 23421-I2C | AGCAGTAGTGGCTGGTGGATTAG | |  | 1059 |  |  |
| 900-o1 | TGGCCCAACAGGAAGAATCAAC | Contig900 | 1425 | 108 | 1218 | CBM10/CE15 |
| 900-o2C | TTAGCCGATTGGGAATGCAGAC |  |  | 1326 |  |  |
| 900-I1 | ACCGAATCTGTCTTCCACTC |  |  | 490 | 662 |  |
| 900-I1c | CCTAGTGGTAATGGTCCATTCC |  |  | 1152 |  |  |
| 28807-o1 | GTGGAGCGACTAAAGCAGTAAG | Contig28807 | 1617 | 327 | 1194 | CBM10/CE15 |
| 28807-o2c | CTTGGCTACCCATGTTGTG |  |  | 1521 |  |  |
| 28807-I1 | CTAAACGGGAACAACCAG |  |  | 582 | 625 |  |
| 28807-I2c | TGCTCCAACTCCAGATAC |  |  | 1207 |  |  |
| node7061-O2 | CTTGCCTTCCAGCTGTTAATGC | Contig Node 7061 | 960 | 44 | 651 | DUF297/CBM10 |
| node7061-o2c | TCGGTTGGGTAACCGTAAAGAG |  |  | 695 |  |  |
| node7061-I1 | CTTATACGGTGCTACCAAGG |  |  | 168 | 373 |  |
| Node7061-I2c | AACCAGCAGCCATGTTAC |  |  | 541 |  |  |
| 260-I1 | GCCAGAGAAGAAGCTAAAGG | Contig260 | 1229 | 301 | 581 | DUF297 |
| 260-I2c | AGTAGAGGCAGAACCAGAAC |  |  | 882 |  |  |
| 260-O1 | CTGGGCTTTAGGTACTAAGG |  |  | 141 | 763 |  |
| 260-O2c | TAGCAGTGGTAGTGGTCTTC |  |  | 904 |  |  |
| 7694-o1 | GTGATGCTCGCAATCTCTAC | Contig7694 | 1435 | 88 | 587 | PL09 |
| 7694-O2C | CCATCAGCATTAGCACCATAGG |  |  | 675 |  |  |
| 7694-I1 | CCCATTTCCATGGCTGAAATGC |  |  | 114 | 419 |  |
| 7694-I2C | TTGCTCCACTAACCCAGATACC |  |  | 533 |  |  |
