# Supplementary material for: Snapshot of the Eukaryotic Gene Expression in Muskoxen Rumen—A Metatranscriptomic Approach
Source: PLoS One. 2011 May 31;6(5):e20521. doi: 10.1371/journal.pone.0020521 (PMC3105075; doi:10.1371/journal.pone.0020521)
Supplement: Table S3 — Metabolic related KOG/COG groups represented by 5000 or more reads in the metatranscriptomes from Muskoxen rumen eukaryotes. (DOC) [file pone.0020521.s013.doc]

**Table S3.** Metabolic related KOG/COG groups represented by 5000 or more reads in the metatranscriptome from muskoxen rumen eukaryotes.

| **KOG/COG Description** | **Reads Number** | **KOG/COG Category** |
| --- | --- | --- |
| **KOG2670, Enolase** | 60668 | Carbohydrate transport and metabolism |
| **KOG0657, Glyceraldehyde 3-phosphate dehydrogenase** | 58954 | Carbohydrate transport and metabolism |
| KOG0626, Beta-glucosidase, lactase phlorizinhydrolase, and related proteins | 45908 | Carbohydrate transport and metabolism |
| **COG0574, PpsA, Phosphoenolpyruvate synthase/pyruvate phosphate dikinase** | 41292 | Carbohydrate transport and metabolism |
| KOG2099, Glycogen phosphorylase | 28858 | Carbohydrate transport and metabolism |
| **KOG4153, Fructose 1,6-bisphosphate aldolase** | 24624 | Carbohydrate transport and metabolism |
| **KOG1367, 3-phosphoglycerate kinase** | 24163 | Carbohydrate transport and metabolism |
| COG2211, MelB, Na+/melibiose symporter and related transporters | 17403 | Carbohydrate transport and metabolism |
| **KOG0625, Phosphoglucomutase** | 10338 | Carbohydrate transport and metabolism |
| KOG1643, Triosephosphate isomerase | 8573 | Carbohydrate transport and metabolism |
| **KOG1369, Hexokinase** | **7768** | **Carbohydrate transport and metabolism** |
| COG0057, GapA, Glyceraldehyde-3-phosphate dehydrogenase/erythrose-4-phosphate dehydrogenase | 6101 | Carbohydrate transport and metabolism |
| COG2115, XylA, Xylose isomerase | 5791 | Carbohydrate transport and metabolism |
| KOG0372, Serine/threonine specific protein phosphatase involved in glycogen accumulation, PP2A-related | 5660 | Carbohydrate transport and metabolism |
| **KOG3749, Phosphoenolpyruvate carboxykinase** | 66252 | Energy production and conversion |
| **KOG1494, NAD-dependent malate dehydrogenase** | 29529 | Energy production and conversion |
| **KOG1255, Succinyl-CoA synthetase, alpha subunit** | 22008 | Energy production and conversion |
| **COG0281, SfcA, Malic enzyme** | 17260 | Energy production and conversion |
| **KOG2799, Succinyl-CoA synthetase, beta subunit** | 17208 | Energy production and conversion |
| KOG0749, Mitochondrial ADP/ATP carrier proteins | 14650 | Energy production and conversion |
| **KOG1257, NADP+-dependent malic enzyme** | **14255** | **Energy production and conversion** |
| **COG1882, PflD, Pyruvate-formate lyase** | **12898** | **Energy production and conversion** |
| KOG0232, Vacuolar H+-ATPase V0 sector, subunits c/c' | 12765 | Energy production and conversion |
| KOG1352, Vacuolar H+-ATPase V1 sector, subunit A | 10826 | Energy production and conversion |
| KOG0453, Aconitase/homoaconitase (aconitase superfamily) | 9878 | Energy production and conversion |
| KOG1351, Vacuolar H+-ATPase V1 sector, subunit B | 8407 | Energy production and conversion |
| **KOG1447, GTP-specific succinyl-CoA synthetase, beta subunit** | 6336 | Energy production and conversion |
| KOG2189, Vacuolar H+-ATPase V0 sector, subunit a | 5029 | Energy production and conversion |
| KOG2250, Glutamate/leucine/phenylalanine/valine dehydrogenases | 15125 | Amino acid transport and metabolism |
| KOG2467, Glycine/serine hydroxymethyltransferase | 8025 | Amino acid transport and metabolism |
| KOG2448, Dihydroxy-acid dehydratase | 7053 | Amino acid transport and metabolism |
| KOG2263, Methionine synthase II (cobalamin-independent) | 6888 | Amino acid transport and metabolism |
| KOG0053, Cystathionine beta-lyases/cystathionine gamma-synthases | 6162 | Amino acid transport and metabolism |
| COG2873, MET17, O-acetylhomoserine sulfhydrylase | 6133 | Amino acid transport and metabolism |
| KOG2790, Phosphoserine aminotransferase | 6060 | Amino acid transport and metabolism |
| KOG1268, Glucosamine 6-phosphate synthetases, contain amidotransferase and phosphosugar isomerase domains | 7607 | Cell wall/membrane/envelope biogenesis |
| KOG1506, S-adenosylmethionine synthetase | 24216 | Coenzyme transport and metabolism |
| KOG1370, S-adenosylhomocysteine hydrolase | 8926 | Coenzyme transport and metabolism |
| KOG0204, Calcium transporting ATPase | 19494 | Inorganic ion transport and metabolism |
| KOG0693, Myo-inositol-1-phosphate synthase | 25686 | Lipid transport and metabolism |
| KOG0059, Lipid exporter ABCA1 and related proteins, ABC superfamily | 9000 | Lipid transport and metabolism |
| KOG0888, Nucleoside diphosphate kinase | 8411 | Nucleotide transport and metabolism |
| KOG0055, Multidrug/pheromone exporter, ABC superfamily | 5171 | Secondary metabolites biosynthesis, transport and catabolism |
