# Supplementary material for: Snapshot of the Eukaryotic Gene Expression in Muskoxen Rumen—A Metatranscriptomic Approach
Source: PLoS One. 2011 May 31;6(5):e20521. doi: 10.1371/journal.pone.0020521 (PMC3105075; doi:10.1371/journal.pone.0020521)
Supplement: Table S4 — The abundance of contigs coding lignocellulytic enzymes [glycoside hydrolases (GHs), carbohydrate esterases (CEs), pectate lyases (PLs), carbohydrate-binding modules (CBMs), and other related modules] in the muskoxen eukaryotic metatranscriptome (Muskoxen MT) and a comparison of their abundance in our databases of rumen fungal (Ru. Fungi) and rumen protozoal genes (Ru. prot.) as well as different anaerobic bacteria, including Bacteroides fragilis (Bfra), Butyrivibrio proteoclasticus (Bpro), Clostridium thermocellum (Cthe), Fibrobacter succinogenes (Fsuc), Prevotella ruminicola (Prum), Ruminococcus flavifaciens (Rfla), and the rumen fungus Piromyces sp. E2 ESTs (Pir. ESTs). (DOC) [file pone.0020521.s014.doc]

**Table S4.** The abundance of contigs coding lignocellulytic enzymes [glycoside hydrolases (GHs), carbohydrate esterases (CEs), pectate lyases (PLs), carbohydrate-binding modules (CBMs), and other related modules] in the muskoxen eukaryotic metatranscriptome (Muskoxen MT) and a comparison of their abundance in our databases of rumen fungal (Ru. Fungi) and rumen protozoal genes (Ru. prot.) as well as different anaerobic bacteria, including *Bacteroides fragilis* (Bfra), *Butyrivibrio proteoclasticus* (Bpro), *Clostridium thermocellum* (Cthe), *Fibrobacter succinogenes* (Fsuc), *Prevotella ruminicola* (Prum), *Ruminococcus flavofaciens* (Rfla), and the rumen fungus *Piromyces* sp. E2 ESTs (Pir. ESTs).

| **CAZY family** | **Pfam Accession** | **Muskoxen MT Contigs all sizes** | **Muskoxen MT Contigs ≥500bp** | **Muskoxen MT Reads** | **Pir. ESTs** | **Ru. fungi** | **Ru. prot** | **Bfra** | | **Bpro** | | **Cthe** | | **Fsuc** | | **Prum** | | | **Rfla** |
| --- | --- | --- | --- | --- | --- | --- | --- | --- | --- | --- | --- | --- | --- | --- | --- | --- | --- | --- | --- |
| ***Glycoside hydrolase catalytic domains*** | | | | | |  |  | |  | |  | |  | |  | |  |  | |
| GH1 | PF00232.11 | 24 | 17 | 32628 | 32 | 5 | 0 | 0 | | 1 | | 2 | | 0 | | 0 | | | 0 |
| GH2 | PF02836.10 | 12 | 10 | 186 | 0 | 0 | 0 | 15 | | 8 | | 1 | | 2 | | 8 | | | 1 |
| GH3 | PF00933.14 | 50 | 22 | 4967 | 4 | 1 | 0 | 10 | | 10 | | 2 | | 3 | | 12 | | | 6 |
| GH4 | PF02056.9 | 0 | 0 | 2 | 0 | 0 | 0 | 0 | | 0 | | 0 | | 0 | | 0 | | | 0 |
| GH5 | PF00150.11 | 75 | 46 | 2423 | 9 | 13 | 13 | 0 | | 5 | | 11 | | 12 | | 5 | | | 11 |
| GH6 | PF01341.10 | 55 | 31 | 26950 | 20 | 27 | 0 | 0 | | 0 | | 0 | | 0 | | 0 | | | 0 |
| GH7 | PF00840.13 | 0 | 0 | 0 | 0 | 0 | 0 | 0 | | 0 | | 0 | | 0 | | 0 | | | 0 |
| GH8 | PF01270.10 | 6 | 6 | 496 | 1 | 0 | 0 | 0 | | 1 | | 2 | | 6 | | 1 | | | 0 |
| GH9 | PF00759.12 | 52 | 42 | 10894 | 22 | 3 | 2 | 0 | | 3 | | 16 | | 9 | | 1 | | | 12 |
| GH10 | PF00331.13 | 71 | 29 | 5658 | 1 | 2 | 7 | 0 | | 6 | | 5 | | 7 | | 3 | | | 6 |
| GH11 | PF00457.10 | 45 | 33 | 22294 | 13 | 36 | 4 | 0 | | 0 | | 1 | | 3 | | 0 | | | 8 |
| GH12 | PF01670.9 | 0 | 0 | 0 | 0 | 0 | 0 | 0 | | 0 | | 0 | | 0 | | 0 | | | 0 |
| GH13 | PF00128.17 | 77 | 47 | 4564 | 1 | 0 | 2 | 6 | | 14 | | 2 | | 3 | | 5 | | | 4 |
| GH14 | PF01373.10 | 5 | 0 | 144 | 0 | 0 | 0 | 0 | | 0 | | 0 | | 0 | | 0 | | | 0 |
| GH15 | PF00723.14 | 0 | 0 | 0 | 0 | 0 | 0 | 0 | | 0 | | 1 | | 0 | | 0 | | | 0 |
| GH16 | PF00722.14 | 17 | 10 | 1797 | 0 | 5 | 0 | 6 | | 2 | | 2 | | 4 | | 2 | | | 3 |
| GH17 | PF00322.10 | 0 | 0 | 0 | 0 | 0 | 0 | 0 | | 0 | | 0 | | 0 | | 0 | | | 0 |
| GH18 | PF00704.21 | 30 | 20 | 1905 | 11 | 0 | 0 | 2 | | 1 | | 4 | | 2 | | 1 | | | 1 |
| **CAZY family** | **Pfam Accession** | **Muskoxen MT Contigs all sizes** | **Muskoxen MT Contigs ≥500bp** | **Muskoxen MT Reads** | **Pir. ESTs** | **Ru. fungi** | **Ru. prot** | **Bfra** | | **Bpro** | | **Cthe** | | **Fsuc** | | **Prum** | | | **Rfla** |
| GH19 | PF00182.12 | 2 | 0 | 8 | 0 | 0 | 0 | 0 | | 0 | | 0 | | 0 | | 0 | | | 0 |
| GH20 | PF00728.15 | 1 | 0 | 61 | 0 | 0 | 0 | 12 | | 0 | | 0 | | 0 | | 2 | | | 0 |
| GH21 | deleted family |  |  |  |  |  |  |  | |  | |  | |  | |  | | |  |
| GH22 | PF00062.13 | 0 | 0 | 1 | 0 | 0 | 0 | 0 | | 0 | | 0 | | 0 | | 0 | | | 0 |
| GH23 | PF01464.13 | 4 | 1 | 105 | 0 | 0 | 0 | 3 | | 0 | | 2 | | 3 | | 3 | | | 0 |
| GH24 | PF00959.12 | 5 | 2 | 3 | 0 | 0 | 0 | 0 | | 0 | | 0 | | 0 | | 1 | | | 1 |
| GH25 | PF01183.13 | 31 | 16 | 2 | 0 | 0 | 0 | 1 | | 5 | | 0 | | 0 | | 3 | | | 7 |
| GH26 | PF02156.8 | 13 | 8 | 1737 | 0 | 6 | 0 | 2 | | 0 | | 3 | | 5 | | 1 | | | 6 |
| GH27 | PF02065.11 | 9 | 6 | 85 | 0 | 0 | 0 | 7 | | 5 | | 0 | | 1 | | 2 | | | 1 |
| GH28 | PF00295.10 | 1 | 0 | 1 | 0 | 0 | 0 | 0 | | 2 | | 0 | | 0 | | 5 | | | 0 |
| GH29 | PF01120.10 | 0 | 0 | 10 | 0 | 0 | 0 | 9 | | 1 | | 0 | | 0 | | 3 | | | 0 |
| GH30 | PF02055.9 | 3 | 3 | 0 | 0 | 0 | 0 | 0 | | 1 | | 2 | | 4 | | 0 | | | 1 |
| GH31 | PF01055.19 | 35 | 4 | 1411 | 5 | 0 | 0 | 4 | | 6 | | 0 | | 0 | | 7 | | | 2 |
| GH32 | PF00251.13 | 9 | 3 | 221 | 0 | 0 | 0 | 3 | | 3 | | 0 | | 0 | | 4 | | | 0 |
| GH33 | PF02012.13 | 4 | 3 | 0 | 0 | 0 | 0 | 5 | | 0 | | 1 | | 1 | | 1 | | | 1 |
| GH34 | PF00064.11 | 0 | 0 | 0 | 0 | 0 | 0 | 0 | | 0 | | 0 | | 0 | | 0 | | | 0 |
| GH35 | PF01301.12 | 0 | 0 | 14 | 0 | 0 | 0 | 4 | | 2 | | 0 | | 0 | | 2 | | | 0 |
| GH36 | PF02065 | 9 | 6 | 85 | 0 | 0 | 0 | 7 | | 5 | | 0 | | 1 | | 2 | | | 1 |
| GH37 | PF01204.11 | 0 | 0 | 28 | 0 | 0 | 0 | 0 | | 0 | | 1 | | 0 | | 0 | | | 0 |
| GH38 | PF01074.15 | 1 | 0 | 80 | 0 | 0 | 0 | 1 | | 1 | | 0 | | 0 | | 1 | | | 0 |
| GH39 | PF01229.10 | 0 | 0 | 2 | 0 | 0 | 0 | 0 | | 1 | | 0 | | 0 | | 0 | | | 0 |
| GH40 | deleted family |  |  |  |  |  |  |  | |  | |  | |  | |  | | |  |
| GH41 | deleted family |  |  |  |  |  |  |  | |  | |  | |  | |  | | |  |
| GH42 | PF02449.8 | 0 | 0 | 2 | 0 | 0 | 0 | 4 | | 3 | | 0 | | 0 | | 2 | | | 1 |
| GH43 | PF04616.7 | 81 | 54 | 139 | 75 | 0 | 0 | 10 | | 13 | | 5 | | 13 | | 18 | | | 9 |
| GH44 | BLAST | 0 | 0 | 5 | 0 | 0 | 0 | 0 | | 0 | | 1 | | 1 | | 0 | | | 1 |
| **CAZY family** | **Pfam Accession** | **Muskoxen MT Contigs all sizes** | **Muskoxen MT Contigs ≥500bp** | **Muskoxen MT Reads** | **Pir. ESTs** | **Ru. fungi** | **Ru. prot** | **Bfra** | | **Bpro** | | **Cthe** | | **Fsuc** | | **Prum** | | | **Rfla** |
| GH45 | PF02015.9 | 58 | 51 | 14034 | 10 | 1 | 0 | 0 | | 0 | | 0 | | 4 | | 0 | | | 0 |
| GH46 | PF01374.11 | 0 | 0 | 0 | 0 | 0 | 0 | 0 | | 0 | | 0 | | 0 | | 0 | | | 0 |
| GH47 | PF01532.13 | 0 | 0 | 51 | 1 | 0 | 0 | 0 | | 0 | | 1 | | 1 | | 0 | | | 0 |
| GH48 | PF02011.8 | 54 | 33 | 106034 | 37 | 2 | 0 | 0 | | 0 | | 2 | | 0 | | 0 | | | 1 |
| GH49 | PF03718.6 | 0 | 0 | 0 | 0 | 0 | 0 | 0 | | 0 | | 0 | | 0 | | 0 | | | 0 |
| GH50 | PB001653 | 0 | 0 | 2 | 0 | 0 | 0 | 0 | | 0 | | 0 | | 0 | | 1 | | | 0 |
| GH51 | PF06964 | 1 | 1 | 112 | 0 | 0 | 0 | 1 | | 2 | | 1 | | 1 | | 7 | | | 0 |
| GH52 | PF03512.6 | 0 | 0 | 0 | 0 | 0 | 0 | 0 | | 0 | | 0 | | 0 | | 0 | | | 0 |
| GH53 | PF07745.6 | 4 | 0 | 83 | 0 | 0 | 0 | 0 | | 2 | | 1 | | 2 | | 2 | | | 1 |
| GH54 | PF09206.4 | 0 | 0 | 0 | 0 | 0 | 0 | 0 | | 0 | | 0 | | 1 | | 0 | | | 0 |
| GH55 | BLAST | 0 | 0 | 26 | 0 | 0 | 0 | 0 | | 0 | | 1 | | 0 | | 0 | | | 0 |
| GH56 | PF01630.11 | 0 | 0 | 0 | 0 | 0 | 0 | 0 | | 0 | | 0 | | 0 | | 0 | | | 0 |
| GH57 | PF03065.8 | 0 | 0 | 0 | 0 | 0 | 0 | 1 | | 1 | | 0 | | 3 | | 1 | | | 0 |
| GH58 | BLAST | 0 | 0 | 0 | 0 | 0 | 0 | 0 | | 0 | | 0 | | 0 | | 0 | | | 0 |
| GH59 | PF02057.8 | 0 | 0 | 0 | 0 | 0 | 0 | 0 | | 0 | | 0 | | 0 | | 0 | | | 0 |
| GH60 | Deleted family |  |  |  |  |  |  |  | |  | |  | |  | |  | | |  |
| GH61 | PF03443.7 | 0 | 0 | 0 | 0 | 0 | 0 | 0 | | 0 | | 0 | | 0 | | 0 | | | 0 |
| GH62 | PF03664.6 | 0 | 0 | 0 | 0 | 0 | 0 | 0 | | 0 | | 0 | | 0 | | 0 | | | 0 |
| GH63 | PF03200.9 | 0 | 0 | 31 | 0 | 0 | 0 | 0 | | 0 | | 0 | | 0 | | 0 | | | 0 |
| GH64 | PB001434 | 4 | 3 | 593 | 0 | 0 | 0 | 0 | | 0 | | 0 | | 0 | | 0 | | | 0 |
| GH65 | PF03632.8 | 0 | 0 | 14 | 0 | 0 | 0 | 2 | | 0 | | 0 | | 0 | | 0 | | | 0 |
| GH66 | PB003959 | 0 | 0 | 0 | 0 | 0 | 0 | 0 | | 0 | | 0 | | 0 | | 0 | | | 0 |
| GH67 | PF07488.5 | 1 | 1 | 639 | 1 | 0 | 0 | 0 | | 1 | | 0 | | 0 | | 1 | | | 0 |
| GH68 | PF02435.9 | 0 | 0 | 0 | 0 | 0 | 0 | 0 | | 0 | | 0 | | 0 | | 0 | | | 0 |
| GH69 | Deleted: now PL16 |  |  |  |  |  |  |  | |  | |  | |  | |  | | |  |
| **CAZY family** | **Pfam Accession** | **Muskoxen MT Contigs all sizes** | **Muskoxen MT Contigs ≥500bp** | **Muskoxen MT Reads** | **Pir. ESTs** | **Ru. fungi** | **Ru. prot** | **Bfra** | | **Bpro** | | **Cthe** | | **Fsuc** | | **Prum** | | | **Rfla** |
| GH70 | PF02324.9 | 0 | 0 | 1 | 0 | 0 | 0 | 1 | | 0 | | 0 | | 0 | | 0 | | | 0 |
| GH71 | PF03659.7 | 0 | 0 | 0 | 0 | 0 | 0 | 0 | | 0 | | 0 | | 0 | | 0 | | | 0 |
| GH72 | PF03198.7 | 5 | 0 | 129 | 0 | 0 | 0 | 0 | | 0 | | 0 | | 0 | | 0 | | | 0 |
| GH73 | PF01832.13 | 2 | 2 | 40 | 0 | 0 | 0 | 1 | | 0 | | 0 | | 0 | | 1 | | | 0 |
| GH74 | BLAST | 22 | 6 | 171 | 1 | 0 | 0 | 0 | | 0 | | 1 | | 1 | | 0 | | | 1 |
| GH75 | PF07335.4 | 0 | 0 | 0 | 0 | 0 | 0 | 0 | | 0 | | 0 | | 0 | | 0 | | | 0 |
| GH76 | PF03663.7 | 3 | 1 | 8 | 0 | 0 | 0 | 3 | | 0 | | 1 | | 0 | | 1 | | | 0 |
| GH77 | PF02446.10 | 22 | 8 | 2643 | 0 | 0 | 0 | 1 | | 1 | | 0 | | 1 | | 2 | | | 1 |
| GH78 | PF05592.4 | 5 | 0 | 181 | 0 | 0 | 0 | 3 | | 4 | | 2 | | 0 | | 1 | | | 0 |
| GH79 | PF03662.7 | 0 | 0 | 0 | 0 | 0 | 0 | 0 | | 0 | | 0 | | 0 | | 0 | | | 0 |
| GH80 | BLAST | 0 | 0 | 0 | 0 | 0 | 0 | 0 | | 0 | | 0 | | 0 | | 0 | | | 0 |
| GH81 | PF03639.6 | 0 | 0 | 0 | 0 | 0 | 0 | 0 | | 0 | | 1 | | 0 | | 0 | | | 0 |
| GH82 | BLAST | 0 | 0 | 0 | 0 | 0 | 0 | 0 | | 0 | | 0 | | 0 | | 0 | | | 0 |
| GH83 | PF00423.12 | 0 | 0 | 0 | 0 | 0 | 0 | 0 | | 0 | | 0 | | 0 | | 0 | | | 0 |
| GH84 | PF07555.6 | 3 | 1 | 182 | 0 | 0 | 0 | 1 | | 0 | | 0 | | 0 | | 0 | | | 0 |
| GH85 | PF03644.6 | 0 | 0 | 0 | 0 | 0 | 0 | 0 | | 0 | | 0 | | 0 | | 0 | | | 0 |
| GH86 | BLAST | 0 | 0 | 0 | 0 | 0 | 0 | 0 | | 0 | | 0 | | 0 | | 0 | | | 0 |
| GH87 | BLAST | 7 | 3 | 433 | 0 | 0 | 0 | 0 | | 0 | | 0 | | 0 | | 0 | | | 0 |
| GH88 | PF07470.6 | 9 | 1 | 45 | 0 | 0 | 0 | 3 | | 5 | | 0 | | 2 | | 2 | | | 1 |
| GH89 | PF05089.5 | 15 | 6 | 1191 | 0 | 0 | 0 | 1 | | 0 | | 0 | | 0 | | 1 | | | 0 |
| GH90 | PF09251 | 0 | 0 | 0 | 0 | 0 | 0 | 0 | | 0 | | 0 | | 0 | | 0 | | | 0 |
| GH90 | BLAST | 0 | 0 | 0 | 0 | 0 | 0 | 0 | | 0 | | 0 | | 0 | | 0 | | | 0 |
| GH91 | BLAST | 0 | 0 | 0 | 0 | 0 | 0 | 0 | | 0 | | 0 | | 0 | | 0 | | | 0 |
| GH92 | PF07971.5 | 0 | 0 | 12 | 0 | 0 | 0 | 8 | | 0 | | 0 | | 0 | | 8 | | | 0 |
| GH93 | BLAST | 0 | 0 | 0 | 0 | 0 | 0 | 0 | | 0 | | 0 | | 0 | | 0 | | | 0 |
| GH94 | BLAST | 2 | 1 | 4308 | 0 | 0 | 0 | 0 | | 1 | | 3 | | 1 | | 1 | | | 2 |
| **CAZY family** | **Pfam Accession** | **Muskoxen MT Contigs all sizes** | **Muskoxen MT Contigs ≥500bp** | **Muskoxen MT Reads** | **Pir. ESTs** | **Ru. fungi** | **Ru. prot** | **Bfra** | | **Bpro** | | **Cthe** | | **Fsuc** | | **Prum** | | | **Rfla** |
| GH96 | BLAST | 0 | 0 | 0 | 0 | 0 | 0 | 0 | | 0 | | 0 | | 0 | | 0 | | | 0 |
| GH97 | PF10566.2 | 2 | 2 | 16 | 0 | 0 | 0 | 4 | | 0 | | 0 | | 0 | | 6 | | | 3 |
| GH98 | PF08306.4 | 0 | 0 | 1 | 0 | 0 | 0 | 0 | | 0 | | 0 | | 0 | | 0 | | | 0 |
| GH99 | PB000173 | 0 | 0 | 12 | 0 | 0 | 0 | 0 | | 0 | | 0 | | 0 | | 1 | | | 0 |
| GH100 | PF04853.5 | 0 | 0 | 1 | 0 | 0 | 0 | 0 | | 0 | | 0 | | 0 | | 0 | | | 0 |
| GH101 | BLAST | 0 | 0 | 0 | 0 | 0 | 0 | 0 | | 0 | | 0 | | 0 | | 0 | | | 0 |
| GH102 | PF03562.10 | 0 | 0 | 1 | 0 | 0 | 0 | 0 | | 0 | | 0 | | 0 | | 0 | | | 0 |
| GH103 | BLAST | 0 | 0 | 1 | 0 | 0 | 0 | 0 | | 0 | | 0 | | 0 | | 0 | | | 0 |
| GH104 | PF00959.12 | 5 | 2 | 3 | 0 | 0 | 0 | 0 | | 0 | | 0 | | 0 | | 1 | | | 1 |
| GH105 | PF07470.6 | 9 | 1 | 45 | 0 | 0 | 0 | 3 | | 5 | | 0 | | 2 | | 2 | | | 1 |
| GH106 | PB000239 | 0 | 0 | 1 | 0 | 0 | 0 | 0 | | 0 | | 0 | | 0 | | 1 | | | 0 |
| GH107 | BLAST | 0 | 0 | 0 | 0 | 0 | 0 | 0 | | 0 | | 0 | | 0 | | 0 | | | 0 |
| GH108 | PF05838.5 | 0 | 0 | 0 | 0 | 0 | 0 | 0 | | 0 | | 0 | | 0 | | 0 | | | 0 |
| GH109 | PF01408 | 2 | 0 | 51 | 1 | 0 | 0 | 5 | | 6 | | 3 | | 3 | | 4 | | | 1 |
| GH110 | PB004422 | 0 | 0 | 0 | 0 | 0 | 0 | 2 | | 0 | | 0 | | 0 | | 0 | | | 0 |
| GH111 | PWGH111 | 0 | 0 | 0 | 0 | 0 | 0 | 0 | | 0 | | 0 | | 0 | | 0 | | | 0 |
| GH112 | PF09508 | 0 | 0 | 1 | 0 | 0 | 0 | 0 | | 1 | | 0 | | 0 | | 0 | | | 0 |
| GH113 | PB000085 | 0 | 0 | 0 | 0 | 0 | 0 | 0 | | 0 | | 0 | | 0 | | 0 | | | 0 |
| GH114 | PF03537 | 23 | 11 | 343 | 38 | 0 | 0 | 0 | | 0 | | 0 | | 0 | | 0 | | | 0 |
| GH115 | PB000184 | 13 | 10 | 1541 | 0 | 0 | 0 | 1 | | 3 | | 0 | | 0 | | 1 | | | 0 |
| GH116 | PF12215 | 0 | 0 | 0 | 0 | 0 | 0 | 0 | | 0 | | 0 | | 1 | | 0 | | | 0 |
| GH117 | PWGH117 | 16 | 14 | 8 | 0 | 0 | 0 | 5 | | 4 | | 3 | | 4 | | 8 | | | 2 |
| GH118 | PWGH118 | 0 | 0 | 0 | 0 | 0 | 0 | 0 | | 0 | | 0 | | 0 | | 0 | | | 0 |
| ***CBMs*** |  |  |  |  |  |  |  |  | |  | |  | |  | |  | | |  |
| CBM1 | PF00734.11 | 85 | 33 | 3697 | 20 | 11 | 0 | 0 | | 0 | | 0 | | 0 | | 0 | | | 0 |
| CBM2 | PF00553.12 | 0 | 0 | 2 | 0 | 0 | 0 | 0 | | 7 | | 0 | | 0 | | 0 | | | 0 |
| **CAZY family** | **Pfam Accession** | **Muskoxen MT Contigs all sizes** | **Muskoxen MT Contigs ≥500bp** | **Muskoxen MT Reads** | **Pir. ESTs** | **Ru. fungi** | **Ru. prot** | **Bfra** | | **Bpro** | | **Cthe** | | **Fsuc** | | **Prum** | | | **Rfla** |
| CBM3 | PF00942.11 | 3 | 3 | 1 | 0 | 0 | 2 | 0 | | 1 | | 20 | | 0 | | 0 | | | 3 |
| CBM4 | PF02018.10 | 5 | 3 | 0 | 0 | 0 | 3 | 2 | | 0 | | 8 | | 5 | | 4 | | | 19 |
| CBM5 | PF02839.7 | 0 | 0 | 1 | 0 | 0 | 0 | 0 | | 0 | | 0 | | 0 | | 0 | | | 0 |
| CBM6 | PF03422.8 | 40 | 27 | 84 | 0 | 1 | 0 | 3 | | 3 | | 15 | | 30 | | 2 | | | 6 |
| CBM7 | deleted entry |  |  |  |  |  |  |  | |  | |  | |  | |  | | |  |
| CBM8 | BLAST | 0 | 0 | 0 | 0 | 0 | 0 | 0 | | 0 | | 0 | | 0 | | 0 | | | 0 |
| CBM9 | PF02018.10 | 5 | 3 | 0 | 0 | 0 | 3 | 2 | | 0 | | 8 | | 5 | | 4 | | | 19 |
| CBM10 | PF02013.9 | 908 | 403 | 100192 | 183 | 84 | 0 | 0 | | 0 | | 0 | | 0 | | 0 | | | 0 |
| CBM11 | PF03425.6 | 0 | 0 | 0 | 0 | 1 | 0 | 0 | | 0 | | 1 | | 6 | | 0 | | | 0 |
| CBM12 | PF02839.7 | 0 | 0 | 1 | 0 | 0 | 0 | 0 | | 0 | | 0 | | 0 | | 0 | | | 0 |
| CBM13 | PF00652.15 | 40 | 31 | 5 | 2 | 0 | 1 | 0 | | 3 | | 1 | | 0 | | 0 | | | 8 |
| CBM14 | PF01607.17 | 0 | 0 | 0 | 0 | 0 | 0 | 0 | | 0 | | 0 | | 0 | | 0 | | | 0 |
| CBM15 | PF03426.7 | 0 | 0 | 0 | 0 | 0 | 0 | 0 | | 0 | | 0 | | 0 | | 0 | | | 0 |
| CBM16 | PF02018.10 | 5 | 3 | 0 | 0 | 0 | 3 | 2 | | 0 | | 8 | | 5 | | 4 | | | 19 |
| CBM17 | PF03424.7 | 0 | 0 | 0 | 0 | 0 | 0 | 0 | | 0 | | 0 | | 0 | | 0 | | | 0 |
| CBM18 | PF00187.12 | 370 | 108 | 22914 | 88 | 0 | 0 | 0 | | 0 | | 0 | | 0 | | 0 | | | 0 |
| CBM19 | PF03427.6 | 0 | 0 | 0 | 0 | 0 | 0 | 0 | | 0 | | 0 | | 0 | | 0 | | | 0 |
| CBM20 | PF00686.12 | 6 | 5 | 84 | 0 | 0 | 0 | 1 | | 0 | | 0 | | 0 | | 2 | | | 0 |
| CBM21 | PF03370.6 | 1 | 0 | 52 | 3 | 0 | 0 | 0 | | 0 | | 0 | | 0 | | 0 | | | 0 |
| CBM22 | PF02018.10 | 5 | 3 | 0 | 0 | 0 | 3 | 2 | | 0 | | 8 | | 5 | | 4 | | | 19 |
| CBM23 | BLAST | 0 | 0 | 0 | 0 | 0 | 0 | 0 | | 0 | | 0 | | 0 | | 0 | | | 0 |
| CBM24 | BLAST | 0 | 0 | 0 | 0 | 0 | 0 | 0 | | 0 | | 0 | | 0 | | 0 | | | 0 |
| CBM25 | PF03423.6 | 0 | 0 | 0 | 2 | 0 | 0 | 0 | | 0 | | 2 | | 0 | | 0 | | | 0 |
| CBM26 | PB013554 | 0 | 0 | 4 | 1 | 0 | 0 | 0 | | 1 | | 0 | | 0 | | 0 | | | 0 |
| CBM27 | PF09212.3 | 0 | 0 | 0 | 0 | 0 | 0 | 0 | | 0 | | 0 | | 0 | | 0 | | | 0 |
| CBM28 | PF03424.7 | 0 | 0 | 0 | 0 | 0 | 0 | 0 | | 0 | | 0 | | 0 | | 0 | | | 0 |
| **CAZY family** | **Pfam Accession** | **Muskoxen MT Contigs all sizes** | **Muskoxen MT Contigs ≥500bp** | **Muskoxen MT Reads** | **Pir. ESTs** | **Ru. fungi** | **Ru. prot** | **Bfra** | | **Bpro** | | **Cthe** | | **Fsuc** | | **Prum** | | | **Rfla** |
| CBM29 | BLAST | 13 | 11 | 2318 | 9 | 19 | 0 | 0 | | 0 | | 0 | | 0 | | 0 | | | 0 |
| CBM30 | BLAST | 0 | 0 | 29 | 0 | 0 | 0 | 0 | | 0 | | 0 | | 3 | | 0 | | | 0 |
| CBM31 | PF11606 | 0 | 0 | 0 | 0 | 0 | 0 | 0 | | 0 | | 0 | | 0 | | 0 | | | 0 |
| CBM32 | PF00754.18 | 6 | 2 | 27 | 0 | 0 | 0 | 21 | | 1 | | 2 | | 1 | | 6 | | | 2 |
| CBM33 | PF03067.8 | 0 | 0 | 0 | 0 | 0 | 0 | 0 | | 0 | | 0 | | 0 | | 0 | | | 0 |
| CBM34 | PF02903.7 | 0 | 0 | 0 | 0 | 0 | 0 | 0 | | 2 | | 1 | | 0 | | 0 | | | 0 |
| CBM35 | BLAST | 0 | 1 | 1 | 0 | 5 | 0 | 0 | | 0 | | 1 | | 1 | | 0 | | | 4 |
| CBM36 | BLAST | 21 | 8 | 0 | 0 | 0 | 0 | 1 | | 1 | | 10 | | 1 | | 2 | | | 2 |
| CBM37 | BLAST | 4 | 3 | 5 | 0 | 0 | 2 | 0 | | 0 | | 2 | | 0 | | 0 | | | 15 |
| CBM38 | BLAST | 0 | 0 | 0 | 0 | 0 | 0 | 0 | | 0 | | 0 | | 0 | | 0 | | | 0 |
| CBM39 | BLAST | 0 | 0 | 0 | 0 | 0 | 0 | 0 | | 0 | | 0 | | 0 | | 0 | | | 0 |
| CBM40 | PF02973.9 | 0 | 0 | 0 | 0 | 0 | 0 | 0 | | 0 | | 0 | | 0 | | 0 | | | 0 |
| CBM41 | PF03714.7 | 0 | 0 | 0 | 0 | 0 | 0 | 0 | | 1 | | 0 | | 0 | | 0 | | | 0 |
| CBM42 | PF05270.6 | 0 | 0 | 4 | 0 | 0 | 0 | 0 | | 0 | | 4 | | 0 | | 0 | | | 0 |
| CBM43 | PF07983.6 | 0 | 0 | 1 | 0 | 0 | 0 | 0 | | 0 | | 0 | | 0 | | 0 | | | 0 |
| CBM44 | BLAST | 0 | 0 | 0 | 0 | 0 | 0 | 0 | | 0 | | 0 | | 0 | | 0 | | | 0 |
| CBM45 | BLAST | 0 | 0 | 0 | 0 | 0 | 0 | 0 | | 0 | | 0 | | 0 | | 0 | | | 0 |
| CBM46 | PF03442 | 0 | 0 | 0 | 0 | 0 | 0 | 0 | | 0 | | 0 | | 0 | | 0 | | | 0 |
| CBM47 | PF00754.18 | 6 | 2 | 27 | 0 | 0 | 0 | 21 | | 1 | | 2 | | 1 | | 6 | | | 2 |
| CBM48 | PF02922.11 | 6 | 3 | 98 | 7 | 0 | 0 | 2 | | 4 | | 1 | | 2 | | 4 | | | 2 |
| CBM49 | PF09478.3 | 0 | 0 | 0 | 0 | 0 | 0 | 0 | | 2 | | 0 | | 0 | | 0 | | | 0 |
| CBM50 | PF01476.13 | 18 | 11 | 1520 | 3 | 0 | 0 | 4 | | 3 | | 10 | | 3 | | 5 | | | 0 |
| CBM51 | PF08305.4 | 0 | 0 | 0 | 0 | 0 | 0 | 0 | | 0 | | 0 | | 3 | | 0 | | | 0 |
| CBM52 | PF10645 | 4 | 1 | 101 | 0 | 0 | 0 | 0 | | 0 | | 0 | | 0 | | 0 | | | 0 |
| CBM53 | BLAST | 0 | 0 | 0 | 0 | 0 | 0 | 0 | | 0 | | 0 | | 0 | | 0 | | | 0 |
| CBM54 | PWCBM54 | 0 | 0 | 0 | 0 | 0 | 0 | 0 | | 0 | | 1 | | 0 | | 0 | | | 0 |
| **CAZY family** | **Pfam Accession** | **Muskoxen MT Contigs all sizes** | **Muskoxen MT Contigs ≥500bp** | **Muskoxen MT Reads** | **Pir. ESTs** | **Ru. fungi** | **Ru. prot** | **Bfra** | | **Bpro** | | **Cthe** | | **Fsuc** | | **Prum** | | | **Rfla** |
| CBM55 | PWCBM55 | 0 | 0 | 0 | 0 | 0 | 0 | 0 | | 0 | | 0 | | 0 | | 0 | | | 0 |
| CBM56 | PWCBM56 | 0 | 0 | 0 | 0 | 0 | 0 | 0 | | 0 | | 0 | | 0 | | 0 | | | 0 |
| CBM57 | PF11721 | 2 | 0 | 19 | 0 | 0 | 0 | 0 | | 1 | | 0 | | 0 | | 0 | | | 0 |
| CBM58 | PWCBM58 | 0 | 0 | 0 | 0 | 0 | 0 | 0 | | 0 | | 0 | | 0 | | 0 | | | 0 |
| CBM59 | PWCBM59 | 0 | 0 | 0 | 0 | 0 | 0 | 0 | | 0 | | 0 | | 0 | | 0 | | | 0 |
| ***Carbohydrate esterases*** |  |  |  |  |  |  |  |  | |  | |  | |  | |  | | |  |
| CE1 | PF00756 | 17 | 13 | 86 | 0 | 2 | 0 | 4 | | 6 | | 3 | | 1 | | 7 | | | 9 |
| CE2+CE3 | PB002673 | 18 | 6 | 85 | 0 | 3 | 0 | 3 | | 2 | | 1 | | 1 | | 2 | | | 3 |
| CE4 | PF01522.14 | 78 | 50 | 983 | 4 | 0 | 0 | 2 | | 5 | | 5 | | 3 | | 2 | | | 6 |
| CE5 | PF01083.15 | 0 | 0 | 0 | 0 | 0 | 0 | 0 | | 0 | | 0 | | 0 | | 0 | | | 0 |
| CE6 | PF03629 | 19 | 19 | 17 | 0 | 2 | 0 | 2 | | 1 | | 0 | | 5 | | 3 | | | 0 |
| CE7 | PF05448 | 1 | 0 | 6 | 0 | 0 | 0 | 2 | | 1 | | 1 | | 1 | | 3 | | | 2 |
| CE8 | PF01095.12 | 10 | 3 | 96 | 0 | 0 | 0 | 0 | | 2 | | 1 | | 1 | | 2 | | | 1 |
| CE9 | PF01979.13 | 7 | 3 | 31 | 0 | 0 | 0 | 5 | | 3 | | 6 | | 2 | | 1 | | | 3 |
| CE10 | PF00135 | 15 | 4 | 745 | 10 | 0 | 0 | 1 | | 5 | | 1 | | 1 | | 3 | | | 0 |
| CE11 | PF03331.6 | 0 | 0 | 4 | 0 | 0 | 0 | 1 | | 0 | | 0 | | 1 | | 1 | | | 0 |
| CE12 | PB008046 | 10 | 5 | 553 | 0 | 0 | 0 | 1 | | 3 | | 1 | | 5 | | 4 | | | 3 |
| CE13 | PF03283 | 0 | 0 | 157 | 0 | 0 | 0 | 0 | | 1 | | 0 | | 0 | | 0 | | | 1 |
| CE14 | PF02585.10 | 0 | 0 | 5 | 0 | 0 | 0 | 1 | | 1 | | 1 | | 0 | | 1 | | | 0 |
| CE15 | PWCE015 | 9 | 8 | 4231 | 0 | 0 | 0 | 1 | | 0 | | 0 | | 1 | | 3 | | | 2 |
| CE16 | PWCE016 | 2 | 0 | 3 | 1 | 0 | 0 | 0 | | 0 | | 0 | | 0 | | 0 | | | 0 |
| ***Polysaccharide lyases*** |  |  |  |  |  |  |  |  | |  | |  | |  | |  | | |  |
| PL1 | PF00544.12 | 10 | 6 | 244 | 0 | 0 | 0 | 0 | | 1 | | 2 | | 6 | | 1 | | | 4 |
| PL2 | PF06917 | 0 | 0 | 0 | 0 | 0 | 0 | 0 | | 0 | | 0 | | 0 | | 0 | | | 0 |
| PL3 | PF03211.6 | 10 | 4 | 3660 | 2 | 0 | 0 | 0 | | 0 | | 0 | | 0 | | 0 | | | 0 |
| **CAZY family** | **Pfam Accession** | **Muskoxen MT Contigs all sizes** | **Muskoxen MT Contigs ≥500bp** | **Muskoxen MT Reads** | **Pir. ESTs** | **Ru. fungi** | **Ru. prot** | **Bfra** | | **Bpro** | | **Cthe** | | **Fsuc** | | **Prum** | | | **Rfla** |
| PL4 | PF06045 | 1 | 1 | 0 | 0 | 0 | 0 | 0 | | 0 | | 0 | | 0 | | 0 | | | 0 |
| PL4 | PF09284 | 0 | 0 | 0 | 0 | 0 | 0 | 0 | | 0 | | 0 | | 0 | | 0 | | | 0 |
| PL5 | PF05426.5 | 0 | 0 | 0 | 0 | 0 | 0 | 0 | | 0 | | 0 | | 0 | | 0 | | | 0 |
| PL6 | PWPL006 | 8 | 7 | 0 | 0 | 0 | 0 | 0 | | 0 | | 1 | | 0 | | 0 | | | 0 |
| PL7+PL18 | PF08787 | 0 | 0 | 0 | 0 | 0 | 0 | 0 | | 0 | | 0 | | 0 | | 0 | | | 0 |
| PL8 | PF02278.11 | 0 | 0 | 8 | 0 | 0 | 0 | 1 | | 0 | | 0 | | 0 | | 0 | | | 0 |
| PL9 | QMPL09 | 15 | 8 | 325 | 0 | 0 | 0 | 2 | | 4 | | 2 | | 2 | | 0 | | | 2 |
| PL10 | PF09492 | 0 | 0 | 0 | 0 | 0 | 0 | 0 | | 0 | | 0 | | 1 | | 1 | | | 0 |
| PL11 | PWPL011 | 1 | 1 | 113 | 0 | 0 | 0 | 0 | | 1 | | 1 | | 2 | | 1 | | | 7 |
| PL13 | PWPL013 | 0 | 0 | 0 | 0 | 0 | 0 | 0 | | 0 | | 0 | | 0 | | 0 | | | 0 |
| PL14 | PB002765 | 0 | 0 | 0 | 0 | 0 | 0 | 0 | | 0 | | 0 | | 1 | | 0 | | | 0 |
| PL16 | PF07212 | 0 | 0 | 0 | 0 | 0 | 0 | 0 | | 0 | | 0 | | 0 | | 0 | | | 0 |
| PL19 | Deleted:NowGH91 |  |  |  |  |  |  |  | |  | |  | |  | |  | | |  |
| PL20 | PWPL020 | 0 | 0 | 0 | 0 | 0 | 0 | 0 | | 0 | | 0 | | 0 | | 0 | | | 0 |
| PL22 | PB009195 | 0 | 0 | 0 | 0 | 0 | 0 | 5 | | 0 | | 0 | | 2 | | 3 | | | 0 |
| PL12+15+17+21 | PF07940 | 0 | 0 | 3 | 0 | 0 | 0 | 2 | | 1 | | 0 | | 0 | | 1 | | | 0 |
|  | | | | |  |  |  |  | |  | |  | |  | |  | | |  |
| ***Other domains associated with GH catalytic or carbohydrate binding domains*** | | | | |  |  |  |  | |  | |  | |  | |  | | |  |
| AXE1 | PF05448.5 | 1 | 0 | 6 | 0 | 0 | 0 | 2 | | 1 | | 1 | | 1 | | 3 | | | 2 |
| Alpha-amylase_C | PF02806.11 | 2 | 2 | 143 | 4 | 0 | 0 | 1 | | 3 | | 1 | | 1 | | 1 | | | 2 |
| Alpha-L-AF_C | PF06964.5 | 1 | 1 | 112 | 0 | 0 | 0 | 1 | | 2 | | 1 | | 1 | | 7 | | | 0 |
| Alpha-mann_mid | PF09261.4 | 0 | 0 | 15 | 0 | 0 | 0 | 1 | | 1 | | 0 | | 0 | | 1 | | | 0 |
| Bac_rhamnosid_N | PF08531.3 | 0 | 0 | 24 | 0 | 0 | 0 | 2 | | 3 | | 0 | | 0 | | 1 | | | 0 |
| Big_1 | PF02369.9 | 0 | 0 | 0 | 0 | 0 | 0 | 0 | | 0 | | 1 | | 0 | | 0 | | | 0 |
|  |  |  |  |  |  |  |  |  | |  | |  | |  | |  | | |  |
|  |  |  |  |  |  |  |  |  | |  | |  | |  | |  | | |  |
| **CAZY family** | **Pfam Accession** | **Muskoxen MT Contigs all sizes** | **Muskoxen MT Contigs ≥500bp** | **Muskoxen MT Reads** | **Pir. ESTs** | **Ru. fungi** | **Ru. prot** | **Bfra** | | **Bpro** | | **Cthe** | | **Fsuc** | | **Prum** | | | **Rfla** |
| Big_2 | PF02368.11 | 0 | 0 | 29 | 0 | 0 | 0 | 0 | | 2 | | 2 | | 0 | | 4 | | | 5 |
| Big_3 | PF07523.5 | 0 | 0 | 1 | 0 | 0 | 0 | 1 | | 1 | | 0 | | 0 | | 0 | | | 3 |
| Big_4 | PF07532.4 | 0 | 0 | 1 | 0 | 0 | 0 | 0 | | 4 | | 1 | | 0 | | 0 | | | 1 |
| Bgal_small_N | PF02929.10 | 3 | 3 | 24 | 0 | 0 | 0 | 5 | | 1 | | 0 | | 1 | | 2 | | | 1 |
| CBM_X | PF06204.4 | 1 | 1 | 793 | 0 | 0 | 0 | 0 | | 1 | | 2 | | 1 | | 1 | | | 2 |
| CelD_N | PF02927.7 | 0 | 0 | 9 | 0 | 0 | 0 | 0 | | 2 | | 4 | | 6 | | 0 | | | 5 |
| CHB_HEX | PF03173.6 | 0 | 0 | 0 | 0 | 0 | 0 | 0 | | 0 | | 0 | | 0 | | 0 | | | 0 |
| CHB_HEX_C | PF03174.6 | 0 | 0 | 0 | 0 | 0 | 0 | 1 | | 1 | | 0 | | 0 | | 0 | | | 0 |
| ChiC | PF06483.4 | 0 | 0 | 0 | 0 | 0 | 0 | 0 | | 0 | | 0 | | 0 | | 0 | | | 0 |
| ChitinaseA_N | PF08329.3 | 0 | 0 | 0 | 0 | 0 | 0 | 0 | | 0 | | 0 | | 0 | | 0 | | | 0 |
| Cohesin | PF00963.11 | 0 | 0 | 1 | 0 | 0 | 0 | 0 | | 0 | | 8 | | 0 | | 0 | | | 2 |
| Dockerin_1 | PF00404.11 | 0 | 0 | 1 | 0 | 0 | 0 | 0 | | 0 | | 72 | | 0 | | 0 | | | 10 |
| fn3 | PF00041.14 | 0 | 0 | 0 | 1 | 0 | 0 | 2 | | 1 | | 6 | | 3 | | 2 | | | 0 |
| GDE_C | PF06202.7 | 3 | 3 | 357 | 3 | 0 | 0 | 1 | | 0 | | 2 | | 0 | | 1 | | | 0 |
| Glucodextran_B | PF09136.3 | 0 | 0 | 0 | 0 | 0 | 0 | 0 | | 0 | | 0 | | 0 | | 0 | | | 0 |
| Glucodextran_N | PF09137.4 | 0 | 0 | 0 | 0 | 0 | 0 | 0 | | 0 | | 0 | | 0 | | 0 | | | 0 |
| Glyco_hydro_2_N | PF02837.11 | 9 | 7 | 463 | 0 | 0 | 0 | 17 | | 9 | | 2 | | 2 | | 13 | | | 2 |
| Glyco_hydro_2 | PF00703.14 | 6 | 4 | 21 | 0 | 0 | 0 | 15 | | 6 | | 1 | | 2 | | 8 | | | 1 |
| Glyco_hydro_3_C | PF01915.15 | 37 | 23 | 2035 | 2 | 1 | 0 | 8 | | 8 | | 1 | | 1 | | 11 | | | 4 |
| Glyco_hydro_20b | PF02838.8 | 0 | 0 | 5 | 0 | 0 | 0 | 8 | | 0 | | 0 | | 0 | | 2 | | | 0 |
| Glyco_hydro_32C | PF08244.5 | 1 | 0 | 0 | 0 | 0 | 0 | 1 | | 1 | | 0 | | 0 | | 1 | | | 0 |
| Glyco_hydro_38C | PF07748.6 | 0 | 0 | 24 | 0 | 0 | 0 | 1 | | 1 | | 0 | | 0 | | 1 | | | 0 |
| Glyco_hydro_42M | PF08532.3 | 0 | 0 | 1 | 0 | 0 | 0 | 0 | | 1 | | 0 | | 0 | | 0 | | | 1 |
| Glyco_hydro_42C | PF08533.3 | 0 | 0 | 0 | 0 | 0 | 0 | 0 | | 0 | | 0 | | 0 | | 0 | | | 1 |
| Glyco_hydro_65N | PF03636.8 | 0 | 0 | 0 | 0 | 0 | 0 | 1 | | 0 | | 0 | | 0 | | 0 | | | 0 |
| Glyco_hydro_65C | PF03633.8 | 0 | 0 | 1 | 0 | 0 | 0 | 1 | | 0 | | 0 | | 0 | | 0 | | | 0 |
| **CAZY family** | **Pfam Accession** | **Muskoxen MT Contigs all sizes** | **Muskoxen MT Contigs ≥500bp** | **Muskoxen MT Reads** | **Pir. ESTs** | **Ru. fungi** | **Ru. prot** | **Bfra** | | **Bpro** | | **Cthe** | | **Fsuc** | | **Prum** | | | **Rfla** |
| Glyco_hydro_67N | PF03648.7 | 0 | 0 | 0 | 0 | 0 | 0 | 1 | | 0 | | 0 | | 0 | | 2 | | | 0 |
| Glyco_hydro_67C | PF07477.5 | 1 | 1 | 406 | 0 | 0 | 0 | 0 | | 1 | | 0 | | 0 | | 1 | | | 0 |
| Glyco_hydro_98C | PF08307.4 | 0 | 0 | 1 | 0 | 0 | 0 | 0 | | 0 | | 0 | | 0 | | 0 | | | 0 |
| Glyco_transf_36 | PF06165.4 | 1 | 1 | 333 | 0 | 0 | 0 | 0 | | 2 | | 3 | | 1 | | 1 | | | 2 |
| GT36_AF | PF06205.4 | 2 | 1 | 588 | 0 | 0 | 0 | 0 | | 1 | | 2 | | 1 | | 1 | | | 2 |
| He_PIG | PF05345.5 | 0 | 0 | 0 | 0 | 0 | 0 | 1 | | 0 | | 0 | | 0 | | 0 | | | 0 |
| PKD | PF00801.13 | 0 | 0 | 3 | 0 | 0 | 0 | 1 | | 0 | | 2 | | 0 | | 0 | | | 0 |
| TIG | PF01833.17 | 7 | 4 | 9 | 0 | 0 | 0 | 0 | | 0 | | 0 | | 0 | | 2 | | | 0 |
| SusD | PF07980.4 | 0 | 0 | 11 | 0 | 0 | 0 | 59 | | 0 | | 0 | | 0 | | 29 | | | 0 |
| TonB_dep_rec (SusC) | PF00593.17 | 0 | 0 | 4 | 0 | 0 | 0 | 82 | | 0 | | 0 | | 1 | | 41 | | | 0 |
| ***Others*** |  |  |  |  |  |  |  |  | |  | |  | |  | |  | | |  |
| SLH | PF00395 | 0 | 0 | 26 | 0 | 0 | 0 | 0 | | 0 | | 25 | | 0 | | 0 | | | 0 |
| Swollenin | SWOLLEN | 22 | 16 | 7065 | 7 | 0 | 0 | 0 | | 0 | | 0 | | 0 | | 0 | | | 0 |
|  |  |  |  |  |  |  |  |  | |  | |  | |  | |  | | |  |
